# Mitochondrial calcium uptake regulates tumour progression in embryonal rhabdomyosarcoma

**DOI:** 10.1038/s41419-022-04835-4

**Published:** 2022-04-30

**Authors:** Hsin Yao Chiu, Amos Hong Pheng Loh, Reshma Taneja

**Affiliations:** 1 Department of Physiology, Healthy Longevity Translational Research Program, Yong Loo Lin School of Medicine, National University of Singapore, Singapore, 117593 Singapore; 2 VIVA-KKH Paediatric Brain and Solid Tumour Programme, KK Women’s and Children’s Hospital, Singapore, 229899 Singapore

**Keywords:** Paediatric cancer, Sarcoma

## Abstract

Embryonal rhabdomyosarcoma (ERMS) is characterised by a failure of cells to complete skeletal muscle differentiation. Although ERMS cells are vulnerable to oxidative stress, the relevance of mitochondrial calcium homoeostasis in oncogenesis is unclear. Here, we show that ERMS cell lines as well as primary tumours exhibit elevated expression of the mitochondrial calcium uniporter (MCU). MCU knockdown resulted in impaired mitochondrial calcium uptake and a reduction in mitochondrial reactive oxygen species (mROS) levels. Phenotypically, MCU knockdown cells exhibited reduced cellular proliferation and motility, with an increased propensity to differentiate in vitro and in vivo. RNA-sequencing of MCU knockdown cells revealed a significant reduction in genes involved in TGFβ signalling that play prominent roles in oncogenesis and inhibition of myogenic differentiation. Interestingly, modulation of mROS production impacted TGFβ signalling. Our study elucidates mechanisms by which mitochondrial calcium dysregulation promotes tumour progression and suggests that targeting the MCU complex to restore mitochondrial calcium homoeostasis could be a therapeutic avenue in ERMS.

## Introduction

Rhabdomyosarcoma (RMS) is the most prevalent soft-tissue sarcoma in childhood and adolescence [1–3]. Even though RMS cells express myoblast determination protein 1 (MYOD), a master regulator of myogenic differentiation, they exhibit a failure to complete the differentiation programme. The two main subtypes are embryonal rhabdomyosarcoma (ERMS) and alveolar rhabdomyosarcoma (ARMS) that account for approximately 70 and 20%, respectively, of all RMS cases [1–4]. ERMS cells possess a more complex karyotype with a loss of heterozygosity at 11p15.5 and a higher mutation burden compared to ARMS [4, 5]. Mutations in RAS, receptor tyrosine kinase or phosphoinositide-3 kinase (PI3K) complexes are most commonly found in ERMS [2, 5]. These pathways maintain redox balance and energy metabolism for cellular functions [6, 7]. Given these genetic aberrations in ERMS and the importance of mitochondrial function in cancer, a few studies have demonstrated that reactive oxygen species (ROS) production and cellular metabolism are altered in ERMS [8–10]. Upregulation of mitochondrial genes in patient tumours has also been reported [11]. These observations suggest that mitochondrial dysfunction may be important in ERMS oncogenesis. Nevertheless, the role of mitochondrial calcium (Ca^2+^) homoeostasis has not been characterised.

Mitochondrial calcium uniporter (MCU) complex is the main channel responsible for mitochondrial Ca^2+^ uptake and requires inner mitochondrial membrane (IMM) potential for Ca^2+^ to enter the mitochondrial matrix. The MCU complex plays a fundamental role in regulating global Ca^2+^ signalling, redox balance, aerobic metabolism and apoptosis [12, 13]. MCU is the main pore-forming protein. The loss of MCU inhibits mitochondrial Ca^2+^ uptake by approximately 75% [14, 15]. Mitochondrial calcium uptake 1 (MICU1) is the gatekeeper of MCU complex and forms a heterodimer with MICU2 [16, 17]. The MICU1-MICU2 complex prevents mitochondrial Ca^2+^ overload under basal cytosolic Ca^2+^ conditions. MICU1 acts to regulate the threshold of MCU opening and cooperates with MICU2 to activate the channel under high Ca^2+^ concentration [18].

MCU and MICU1 deregulations have been reported in several cancers [13, 19, 20]. For instance, MCU overexpression in breast cancer correlates with tumour size, invasiveness and poor prognosis [21]. In colorectal cancer, MCU-induced mitochondrial Ca^2+^ uptake promotes mitochondrial biogenesis and tumour growth through mitochondrial transcription factor A (TFAM) and nuclear factor kappa-light-chain-enhancer of activated B cells (NF-κB) [22]. The elevation in mitochondrial Ca^2+^ through MCU overexpression promotes hepatocellular carcinoma (HCC) metastasis through ROS production [23]. In contrast, the downregulation of MCU in cervical and colon cancer favours survival [24]. MICU1 expression is deregulated in liver, breast and ovarian cancer. Low MICU1 expression in HCC is correlated with poor prognosis [25], but paradoxically, MICU1 overexpression in ovarian cancer correlates with poor survival and chemoresistance [26, 27]. While the deregulation of expression varies in a cancer type-specific manner, in general, overexpression of MCU and loss of MICU1 expression are correlated with poor prognosis [13, 28].

In this study, we show that MCU is overexpressed in ERMS tumours and its silencing causes a reduction in mitochondrial Ca^2+^ uptake. This is correlated with reduced mitochondrial ROS (mROS) production. Reduction of MCU expression impaired cellular proliferation and motility, while enhancing myogenic differentiation. Interestingly, transforming growth factor beta (TGFβ) signalling pathway was dampened upon MCU knockdown consequent to reduced mROS levels. Elevating mROS reversed the phenotypes observed upon MCU depletion. Our study elucidates the relevance of mitochondrial Ca^2+^ signalling in driving tumour progression.

## Results

### MCU is overexpressed in ERMS

**Fig. 1:**
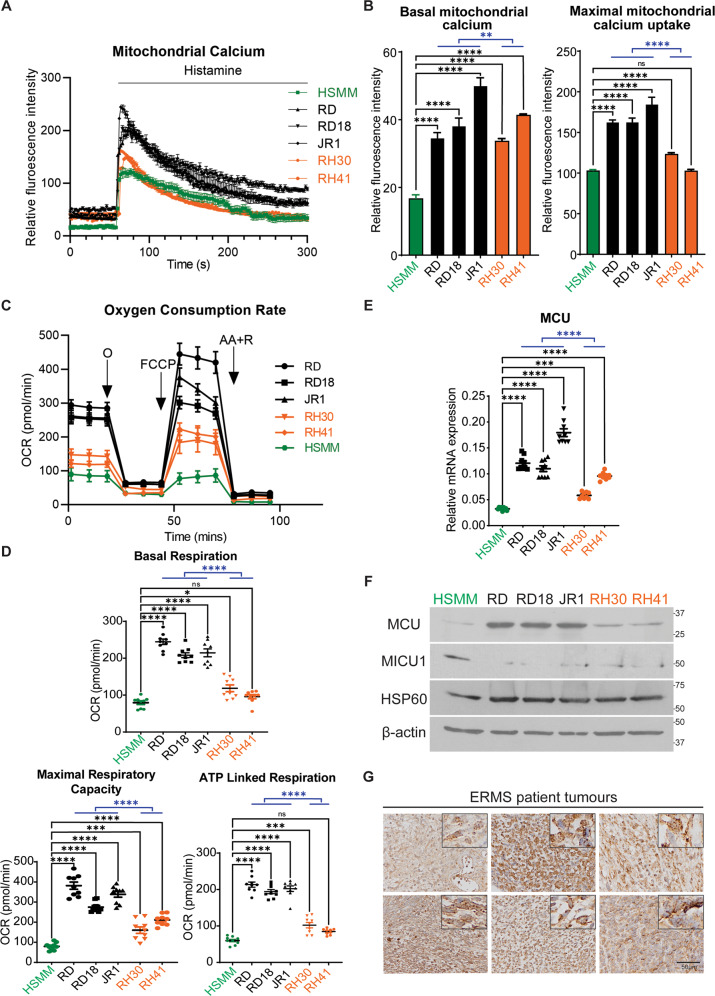
Altered mitochondrial function and MCU expression in ERMS cell lines and patient samples. **A**, **B** Basal and maximal mitochondrial Ca^2+^ levels in HSMM, RD, RD18, JR1, RH30 and RH41 cells was measured with Rhod2-AM staining. The graph on the right below shows basal and maximal mitochondrial Ca^2+^ uptake upon induction with 100 μM histamine (*n* = 3). Values correspond to the average ± SEM. The blue line indicates significance calculated by comparing the average of ERMS cell lines and ARMS cell lines. **C**, **D** Oxygen consumption rate (OCR) was measured with Seahorse analyser with the addition of O: Oligomycin, FCCP: Carbonyl cyanide-4-(trifluoromethoxy)-phenylhydrazone, AA + R: Antimycin A and Rotenone accordingly. Values correspond to average ± SEM (*n* = 3). Basal and maximal respiration rate of HSMM, ERMS and ARMS cell lines as well as mitochondrial ATP-linked respiration are shown (*n* = 3). The blue line shows significance between the average of ERMS cell lines and ARMS cell lines. **E** MCU mRNA was examined in HSMM, RD, RD18, JR1, RH30 and RH41 by qPCR analysis (*n* = 3). Values correspond to average ± SEM. Statistical significance was calculated by one-way ANOVA analysis. The blue line shows the significance comparing the average of ERMS cell lines and ARMS cell lines. **F** Western blot analysis showing MCU, MICU1 and HSP60 protein levels in HSMM, RD, RD18, JR1, RH30 and RH41 cells. β-actin was used as loading control. A representative image of three independent experiments is shown. **G** 6 archival ERMS patient tumour specimens were analysed by IHC using anti-MCU antibody. Images were taken at ×40 magnification. Inset shows ×3 zoomed in image. Scale bar: 50 μm. One-way ANOVA test with appropriate correction was performed for statistical analysis. ns not significant, *p ≤ 0.05, **p ≤ 0.01, ***p ≤ 0.001 and ****p ≤ 0.0001.

Previous studies have suggested deregulation of oxidative stress in ERMS [10, 29, 30]. We therefore examined mitochondrial Ca^2+^ uptake, oxygen consumption rate (OCR) and adenosine triphosphate (ATP)-linked respiration in three patient-derived ERMS cell lines (RD, RD18 and JR1). As controls, we used primary human skeletal muscle myoblasts (HSMM) and two ARMS cell lines (RH30 and RH41). Live-cell staining using Rhod2-AM revealed significantly elevated mitochondrial Ca^2+^ in ERMS cell lines as compared to HSMM and ARMS cell lines (Fig. 1A). The uptake of mitochondrial Ca^2+^ upon induction is crucial in relaying signals. Basal fluorescence was measured for 1 min before Ca^2+^ uptake was induced with 100 μM of histamine. Maximal mitochondrial Ca^2+^ uptake was measured by quantifying the difference between basal fluorescence and highest fluorescence intensity attained post-induction. A significant increase in basal and maximal mitochondrial Ca^2+^ uptake was seen in all three ERMS cell lines relative to HSMM (Fig. 1B). We then analysed mitochondrial function through measurement of OCR and ATP-linked respiration. A significant increase in basal and maximal OCR, as well as in ATP production, were seen in ERMS cell lines relative to HSMM and ARMS cell lines (Fig. 1C, D).

Mitochondrial Ca^2+^ uptake into the inner mitochondrial matrix is tightly regulated by the MCU complex. Therefore, we examined the expression of MCU, the main pore forming subunit of MCU complex in ERMS. MCU was found to be overexpressed in all three ERMS cell lines at both the mRNA and protein level compared to HSMM and ARMS cell lines (Fig. 1E, F). On the other hand, MICU1 expression was downregulated in all three ERMS cell lines (Fig. 1F). No significant difference was observed in the expression Heat Shock Protein 60 (HSP60) a mitochondrial molecular chaperone (Fig. 1F), suggesting that there was no overt change in mitochondrial mass that would account for the change in MCU and MICU1 expression. MCU expression was also examined in six archival ERMS tumour sections by immunohistochemistry (IHC) using anti-MCU antibody (Fig. 1G). All samples showed high MCU expression with varying degrees of speckling. Similarly, a tissue microarray (TMA) of 27 ERMS patient tumours showed elevated MCU expression as compared to 24 ARMS patient samples and 8 normal muscles (Supplementary Fig. 1).

### MCU knockdown decreases mitochondrial function in ERMS cells

**Fig. 2:**
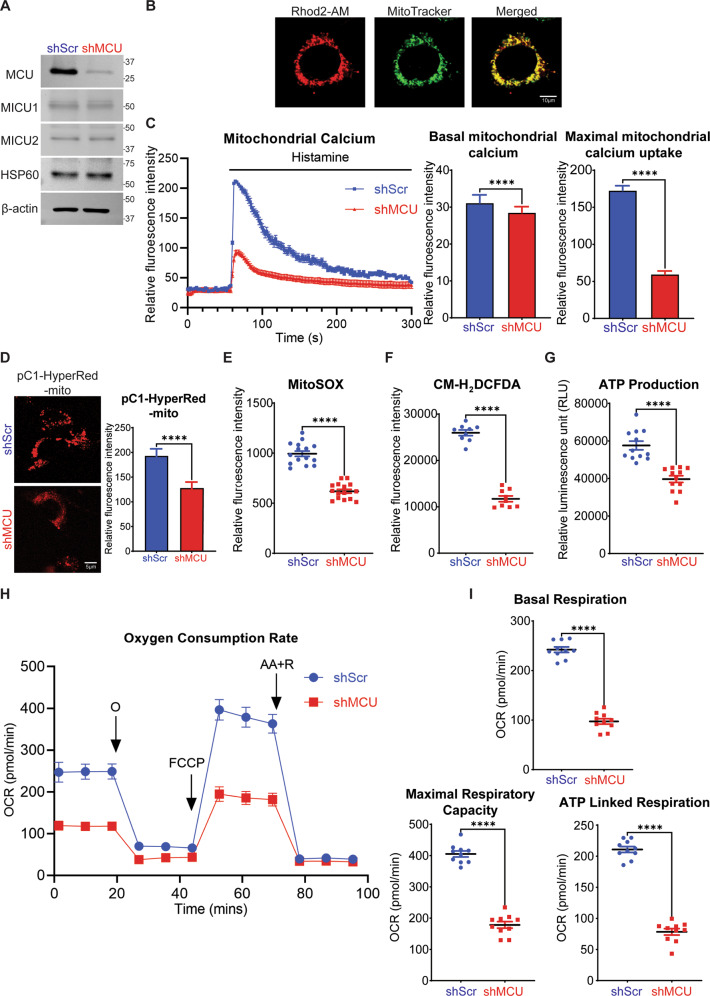
MCU regulates mitochondrial functions in ERMS. **A** Western blot analysis showed significant downregulation of MCU expression in stable MCU knockdown RD cells with no change in MICU1, MICU2 and HSP60 expression. The western blot is representative of three independent experiments. **B**, **C** Co-localisation of Rhod2-AM and MitoTracker staining. Scales bar: 10 µm. Basal and maximal mitochondrial Ca^2+^ uptake upon induction with 100 μM histamine using Rhod2-AM staining is shown in control and shMCU cells (*n* = 3). Values correspond to the average ± SEM. **D** Mitochondrial hydrogen peroxide measurement using pC1-HyperRed-mito fluorescent probe. Scale bar: 5 μm. Fluorescence intensity quantified with Image J (*n* = 3). Values correspond to average ± SEM. **E** MitoSOX Red staining of shMCU in comparison to shScr is shown (*n* = 5). Values correspond to average ± SEM. **F** Cellular ROS in shMCU as compared to shScr was measured by flow cytometry using CM-H_2_DCFDA staining (*n* = 3). Values correspond to average ± SEM. **G** ATPlite kit revealed reduced ATP production in shMCU as compared to shScr (*n* = 4). Values correspond to average ± SEM. **H**, **I** OCR was measured in shMCU cells compared to shScr cells. O: Oligomycin, FCCP: Carbonyl cyanide-4-(trifluoromethoxy)-phenylhydrazone, AA + R: Antimycin A and Rotenone were added accordingly. Values correspond to average ± SEM (*n* = 3). Basal and maximal respiration rate along with ATP-linked respiration in shScr and shMCU cells is shown. Two-tailed non-parametric unpaired *t* test was performed for statistical analysis. ****p ≤ 0.0001.

To investigate the relevance of MCU overexpression, RD cells were transfected with non-targeting control shRNA (shScr) or MCU-specific shRNA (shMCU). The knockdown of MCU was specific, with no change in MICU1, MICU2 and HSP60 levels (Fig. 2A). We next examined the effect of MCU knockdown on mitochondrial Ca^2+^ concentration using Rhod2-AM which localised specifically in the mitochondria as seen by co-localisation with MitoTracker (Fig. 2B). Upon histamine induction, a pronounced 65% reduction in maximal mitochondrial Ca^2+^ uptake was observed in shMCU cells with a small, albeit significant decrease in basal mitochondrial Ca^2+^ (Fig. 2C). A significant reduction in mROS including hydrogen peroxide and superoxide was seen with pC1-HyperRed-mito fluorescent probe (Fig. 2D) and MitoSOX staining (Fig. 2E) respectively. Reduced cytosolic ROS was also observed with CM-H_2_DCFDA staining in shMCU cells (Fig. 2F). Since ATP and mROS are produced by electron transport chain (ETC) during cellular respiration, we examined ATP production and OCR. A significant reduction in overall ATP production was observed in shMCU cells (Fig. 2G). Correspondingly, up to 70% reduction in basal and maximal respiration rate was seen upon MCU knockdown, and ATP-linked respiration through oxidative phosphorylation (OXPHOS) also showed a significant reduction (Fig. 2H, I). Similarly, MCU knockdown in JR1 cells resulted in a significant reduction in maximal mitochondrial Ca^2+^ uptake with no change in basal mitochondrial Ca^2+^ (Supplementary Fig. 2A, B). In addition, a reduction in MitoSOX staining (Supplementary Fig. 2C), basal and maximal respiration rate, as well as ATP-linked respiration was seen upon MCU knockdown (Supplementary Fig. 2D, E).

To further examine the link between MCU and mROS, we overexpressed MCU in C2C12 mouse myoblast cells. MCU overexpression (pMCU) resulted in a significant increase in maximal mitochondrial Ca^2+^ uptake, with no change in basal mitochondrial Ca^2+^ (Supplementary Fig. 3A, B). A significant elevation in mROS production was also observed upon MCU overexpression (Supplementary Fig. 3C). Together these results demonstrate that modulation of MCU expression is sufficient to change mitochondrial function.

### MCU knockdown decreases oncogenic phenotypes in ERMS cells

**Fig. 3:**
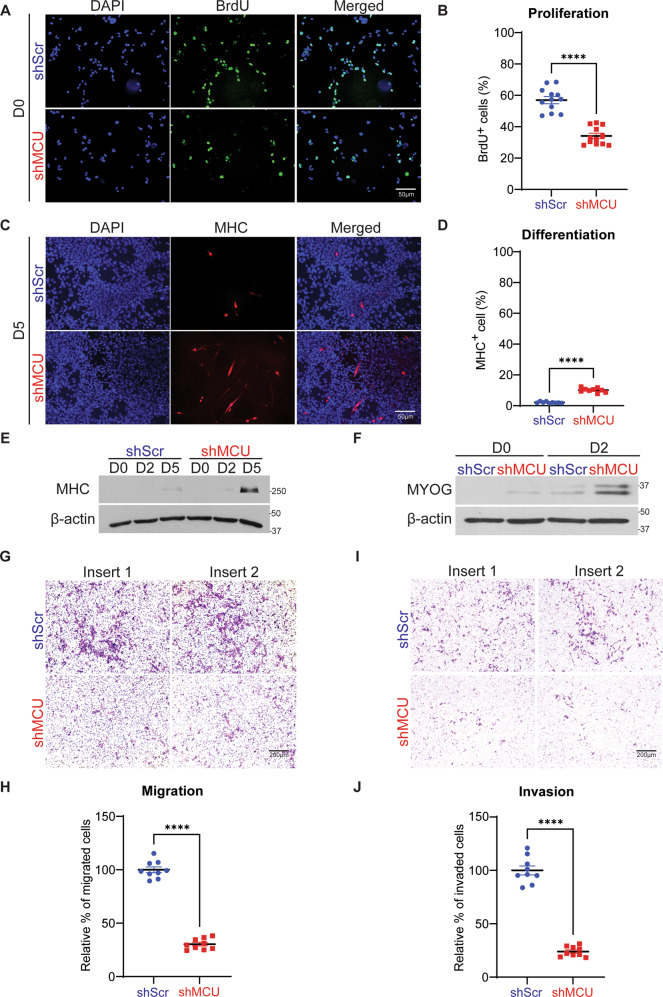
MCU knockdown inhibits oncogenic phenotypes. **A**, **B** BrdU assay to examine proliferation in shScr and shMCU cells. BrdU^+^ cells were analysed by immunofluorescence (*n* = 4). Images are representative of four independent experiments. Scale bar: 50 μm. The scatter plot shows the percentage of BrdU^+^ cells in shMCU cells relative to controls. The values correspond to average ± SEM. **C**, **D** Control shScr and shMCU cells were cultured for 5 days in differentiation medium and analysed by immunofluorescence using anti-MHC antibody. Nuclei were stained with DAPI. Representative images of four independent experiments are shown. Scale bar: 50 μm. The scatter plot shows the percentage of MHC^+^ cells in shMCU cells relative to controls. The values correspond to average ± SEM. **E** MHC level was analysed in control and shMCU cells by western blot analysis at Day 0 (D0), D2 and D5 in differentiation medium. **F** MYOG level was analysed in control and shMCU cells by western blot analysis at D0 and D2. **G**, **H** Boyden chamber migration assay of control and shMCU cells. Migrated cells were observed after 24 h using crystal violet staining. Images are representative of four independent experiments. Scale bar: 200 μm. The relative percentage of migrated cells were quantified in the scatter plot and the values correspond to average ± SEM. **I**, **J** Matrigel invasion assay of control and shMCU cells. Invaded cells were stained with crystal violet after 24 h. Images are representative of four independent experiments. Scale bar: 200 μm. The relative percentage of invaded cells were quantified in the scatter plot and the values correspond to average ± SEM. Two-tailed non-parametric unpaired *t* test was performed for statistical analysis. ****p ≤ 0.0001.

We next examined the phenotype of MCU knockdown cells. A significant reduction in the percentage of 5-bromo-2’-deoxy-uridine-positive (BrdU^+^) cells was seen in shMCU cells relative to control cells (Fig. 3A, B). Similarly, transient MCU knockdown in RD, RD18, JR1 and RH36 cells reduced their proliferative capacity (Supplementary Fig. 4). Conversely, there was an increase in the number of BrdU^+^ cells in MCU-overexpressing cells compared to controls (Supplementary Fig 3E). RD shScr and shMCU cells were differentiated and stained with anti-myosin heavy chain (MHC) antibody. An increase in MHC -positive (MHC^+^) cells was observed in shMCU cells, as well as in siMCU RD, RD18, JR1 and RH36 cells; increase in MHC expression was also verified by western blot analysis (Fig. 3C–E and Supplementary Fig. 5). Myogenin (MYOG), an early myogenic differentiation marker, was also elevated in expression (Fig. 3F). We then investigated the migratory and invasive capacity of shScr and shMCU cells. A profound reduction of approximately 80% in the migratory capacity of shMCU and siMCU cells compared to controls was seen (Fig. 3G, H and Supplementary Fig. 6). MCU knockdown also significantly decreased invasiveness through matrigel (Fig. 3I, J). In contrast to ERMS cell lines, no significant differences were apparent in proliferation, differentiation and migration upon MCU knockdown in the ARMS cell line RH30 (Supplementary Fig. 7A–G).

### TGFβ signalling pathway is downregulated upon MCU knockdown

**Fig. 4:**
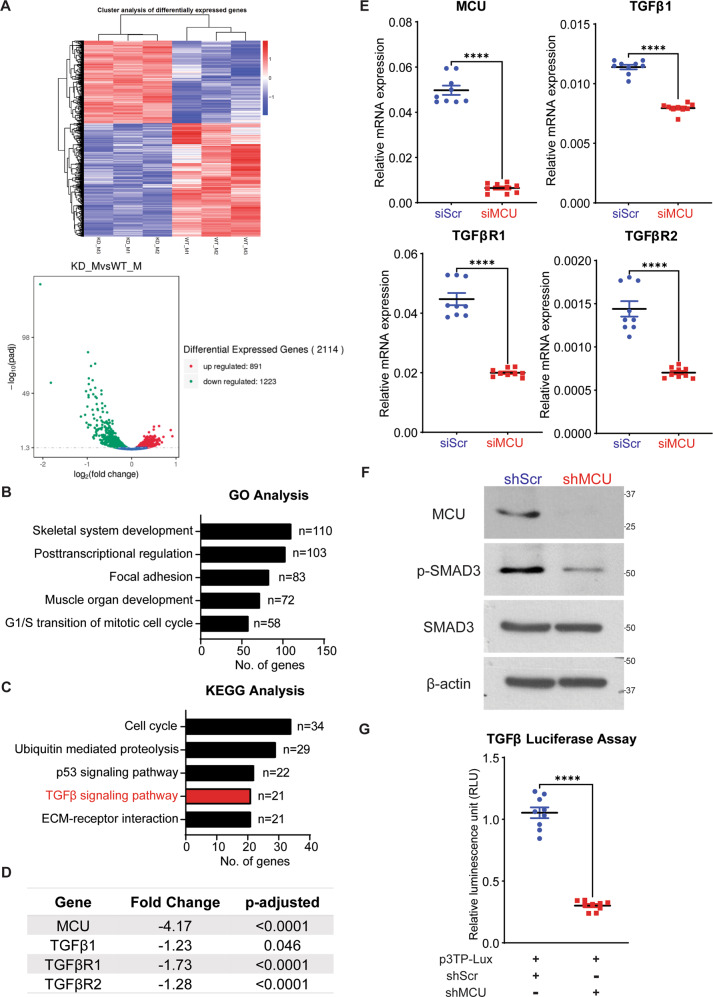
TGFβ signalling pathway is deregulated upon MCU knockdown. **A** RNA-Seq heatmap (upper panel) showing hierarchical cluster of differentially expressed genes. Red represents high expression and blue represents low expression. Volcano plot (lower panel) shows distribution of differentially expressed genes upon MCU knockdown. **B** GO enrichment histogram showing top five significantly enriched biological processes upon MCU knockdown based on the number of differentially expressed genes. **C** KEGG enrichment histogram showing top five unique significantly enriched pathways upon MCU knockdown based on the number of differentially expressed genes. **D** A list of the top significantly altered genes in the TGFβ pathway identified by RNA-Seq analysis upon MCU knockdown are shown with the fold change and adjusted *p* values. **E** qPCR analysis for *TGFβ1*, *TGFβR1* and *TGFβR2* mRNA in control and siMCU cells. The values correspond to average ± SEM (*n* = 3). **F** Western blot analysis of control and shMCU cells using MCU, p-SMAD3 and SMAD3 antibodies. A representative western blot from three independent experiments is shown. **G** Control and shMCU cells were transfected with 200 ng of the TGFβ reporter p3TP-Lux and 5 ng Renilla luciferase. Cells were analysed for luciferase activity 48 h later. The values correspond to average ± SEM (*n* = 3). Two-tailed non-parametric unpaired t-test was performed for statistical analysis. ****p ≤ 0.0001.

In order to identify mechanisms underlying MCU function, we performed RNA-Sequencing (RNA-Seq). Cluster analysis of differentially expressed genes from control RD and MCU knockdown cells, and volcano plot of differentially expressed genes (Fig. 4A) revealed that 891 genes were significantly up regulated and 1223 genes were significantly down regulated in siMCU cells. Gene Ontology (GO) analysis showed that skeletal system development, muscle organ development and focal adhesion were among the top 5 unique biological processes associated with differentially expressed genes in siMCU cells (Fig. 4B and Supplementary Fig. 8A). Kyoto Encyclopaedia of Genes and Genomes (KEGG) pathway analysis identified TGFβ signalling pathway to be among the top 5 significantly altered pathways upon MCU knockdown (Fig. 4C, D and Supplementary Fig. 8B). We validated the downregulation of *TGFβ1*, *TGFβR1* and *TGFβR2* expression by qPCR in MCU knockdown cells (Fig. 4E). Several genes that regulate myogenesis such as myostatin (*MSTN*) [31] and hairy/enhancer-of-split related with YRPW motif protein 2 (*HEY2*) [32] were also downregulated upon MCU knockdown and validated by qPCR (Supplementary Fig. 8C). The differential expression of integrins *ITGB3*, *ITGA3* and *ITGA7* were also validated in shMCU cells (Supplementary Fig. 8D). The TGFβ signalling pathway is well known for its role in tumour progression and epithelial-to-mesenchymal transition (EMT) [33, 34]. We therefore focused on examining this pathway. Consistent with reduced expression of *TGFβ1*, *TGFβR1* and *TGFβR2*, phosphorylated Smad family member 3 (p-SMAD3) levels, a readout of TGFβ signalling, was reduced in shMCU cells whereas total SMAD3 levels were unchanged (Fig. 4F). A significant reduction in the TGFβ reporter 3TP-Lux [35] activity was seen in shMCU cells (Fig. 4G). Consistently, MCU overexpression resulted in a significant increase in TGFβ activity in C2C12 cells as compared to control cells (pCMV) (Supplementary Fig. 3D). Basal TGFβ activity however was not elevated in the ARMS cell lines RH30 and RH41 (Supplementary Fig. 7H). Importantly, several transcriptional targets of the pathway whose expression was altered by RNA-Seq data, including latent transforming growth factor beta binding protein 2 (*LTBP2), LTBP4*, matrix metallopeptidase 16 (*MMP16)*, metalloproteinase inhibitor 3 *(TIMP3)* and serpin family E member 1 (*SERPINE1)* [36–39], were differentially expressed in shMCU cells (Supplementary Fig. 8E). Together, these data demonstrate that elevation of MCU expression is sufficient to modulate TGFβ signalling.

### MCU promotes tumour growth in vivo

**Fig. 5:**
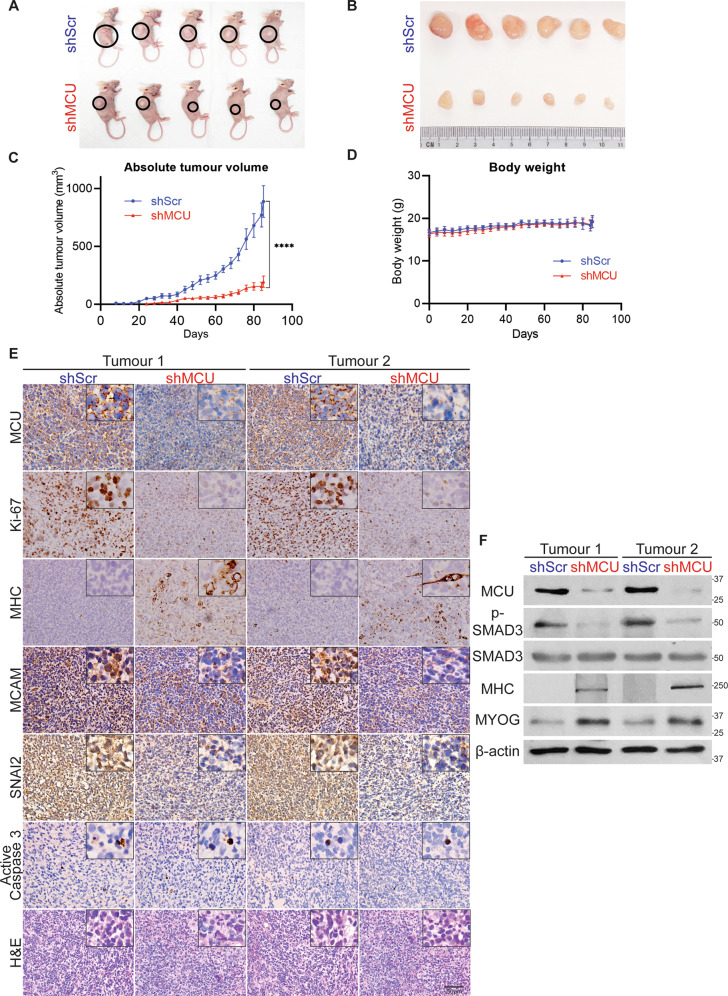
MCU promotes ERMS tumour growth in vivo. **A**, **B** Nude mice were injected with shScr cells (*n* = 7) or shMCU cells (*n* = 7). Representative images of 5 mice in each group (**A**), and resected tumours of 6 mice in each group (**B**) are shown. **C**, **D** The absolute tumour volume and body weight are shown in the graphs below. Statistical significance was calculated using repeated-measure one-way ANOVA where ****p ≤ 0.0001. Values correspond to the average ± SEM. **E** Tumours from two shScr and two shMCU mice were analysed by IHC using anti-MCU, anti-Ki67, anti-MHC, anti-MCAM, anti-SNAI2 and anti-active caspase 3 antibodies. Histology was assessed by haematoxylin and eosin (H&E) staining. Images were taken at ×40 magnification. Inset shows ×3 zoomed in image. Scale bar: 50 μm. **F** Two sets of tumour lysates from shScr and shMCU mice were analysed by western blot with anti-p-SMAD3, anti-SMAD3, anti-MHC, anti-MYOG and anti-β-actin antibodies.

To examine the impact of MCU loss in vivo, we injected RD control (shScr) and shMCU cells in BALB/c nude mice. A significant reduction in tumour growth was apparent in mice injected with shMCU cells (Fig. 5A–C) without any adverse effect on weight (Fig. 5D). Tumour sections from shScr and shMCU cells were analysed histologically and by IHC (Fig. 5E). Ki-67, a proliferation marker, was significantly reduced in shMCU tumours. In contrast, myogenic differentiation was strikingly increased as seen from MHC and MYOG levels by IHC and western blot analysis of tumour lysates. Melanoma Cell Adhesion Molecule (MCAM) and Snail Family Transcriptional Repressor 2 (SNAI2), which promote metastasis and oncogenic progression [40, 41] were decreased. No overt change in active caspase 3 staining was apparent. Moreover, a significant reduction in p-SMAD3 levels was seen in MCU knockdown tumours by western blot analysis (Fig. 5F).

### MCU regulates TGFβ signalling pathway through mROS

**Fig. 6:**
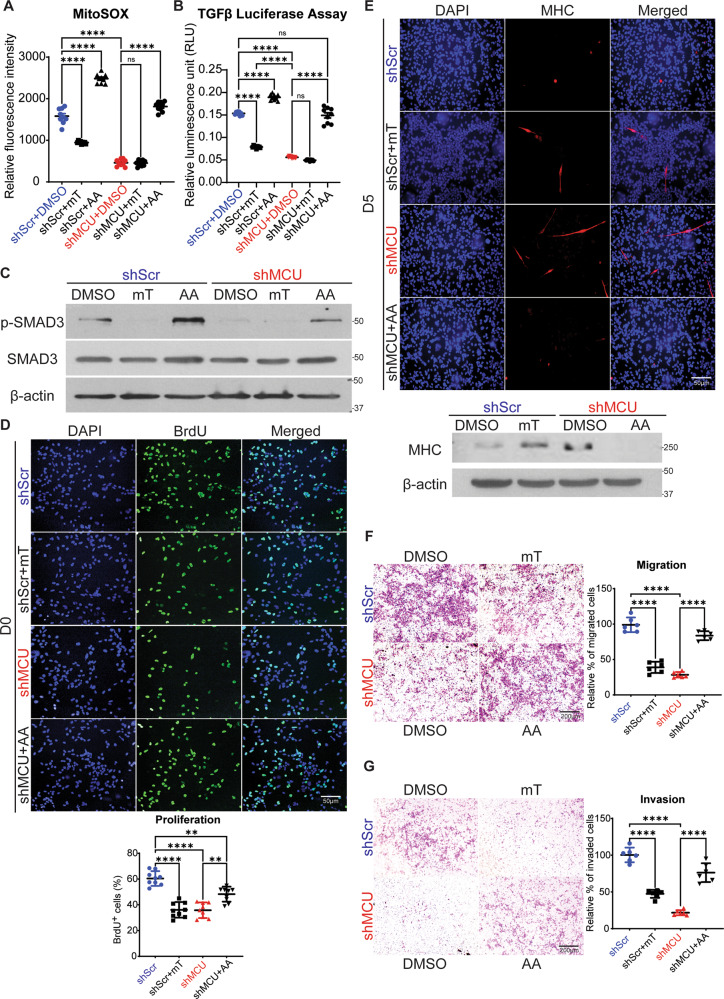
Modulation of mROS impacts TGFβ signalling. **A** shScr and shMCU RD cells were treated with DMSO, mitoTEMPO (mT) or antimycin A (AA) for 48 h. MitoSOX staining showed significantly decreased mROS levels upon mT treatment in shScr cells, whereas increased mROS levels were observed with AA treatment in both shScr and shMCU cells. The values correspond to average ± SEM (*n* = 3). **B** shScr and shMCU cells were treated with mT and AA as indicated for 48 h. Cells were transfected with the p3TP-Lux and analysed for luciferase activity 48 h later. The values correspond to average ± SEM (*n* = 3). **C** p-SMAD3 and SMAD3 levels were examined by western blot analysis in shScr and shMCU cells treated with mT and AA. Representative images of three independent experiments are shown. **D** Proliferation was analysed by BrdU assay in shScr cells and shMCU cells treated with mT or AA for 48 h as indicated. Images are representative of three independent experiments. Scale bar: 50μm. The bar graph shows the percentage of BrdU^+^ cells in shMCU cells relative to shScr cells. The values correspond to average ± SEM. **E** shScr cells were treated for 5 days in differentiation media with DMSO or mT and shMCU cells were treated with DMSO or AA. MHC^+^ cells were analysed by immunofluorescence and quantified using western blot with anti-MHC antibody. Nuclei were stained with DAPI. Representative images of three independent experiments are shown. Scale bar: 50 μm. **F** Migration was analysed for 24 h using Boyden chamber assays following 48 h treatment of shScr cells with DMSO or mT and shMCU cells with DMSO or AA. Images are representative of three independent experiments. Scale bar: 200 μm. The relative percentage of migrated cells were quantified in the scatter plot. The values correspond to average ± SEM.**G** Matrigel invasion was analysed after 24 h following treatment of shScr cells with DMSO or mT and shMCU cells with DMSO or AA for 48 h. Images are representative of three independent experiments. Scale bar: 200 μm. The relative percentage of migrated cells were quantified in the scatter plot and the values correspond to average ± SEM. One-way ANOVA test with appropriate correction was performed for statistical analysis. ns not significant, **p ≤ 0.01, ****p ≤ 0.0001.

Previous studies have shown crosstalk between ROS and TGFβ signalling [42, 43]. We therefore examined whether mROS is upstream of and regulates TGFβ signalling. To alter mROS levels, RD cells were treated with mitoTEMPO (a mROS scavenger) and antimycin A (a complex IV inhibitor) for 48 h. Treatment with mitoTEMPO resulted in a significant reduction in mROS levels in shScr cells, while no further decrease was observed in shMCU cells. On the other hand, treatment with antimycin A elevated mROS levels in both shScr and shMCU cells. (Fig. 6A). MitoTEMPO treatment of shScr cells significantly reduced TGFβ activity to levels similar to shMCU cells, although shMCU cells showed no further reduction in TGFβ activity (Fig. 6B). Consistently, p-SMAD3 level was reduced in mitoTEMPO treated shScr cells with no observable difference in mitoTEMPO-treated shMCU cells (Fig. 6C). Conversely, treatment of shMCU cells with antimycin A rescued TGFβ reporter activity (Fig. 6B) and p-SMAD3 levels to those comparable to control DMSO-treated cells (Fig. 6C).

MCU regulates mROS through increased mitochondrial Ca^2+^ production [44–46]. We first examined whether the phenotypic effects of MCU depletion are mROS-dependent. Upon treatment of control cells with mitoTEMPO, BrdU^+^ cells were reduced to a level similar to shMCU cells. On the other hand, treatment of shMCU cells with antimycin A partially rescued proliferation (Fig. 6D). Treatment with mitoTEMPO also increased the number of MHC^+^ cells in control cells, and conversely, a prominent reduction in MHC staining was seen upon antimycin A treatment in shMCU cells (Fig. 6E). Similarly, migration and invasion were decreased in mitoTEMPO treated shScr cells, whereas increased migration and invasion were observed in antimycin A-treated shMCU cells (Fig. 6F, G).

We next examined the mechanisms by which mROS regulates TGFβ ligand and receptors. *TGFβ1, TGFβR1* and *TGFβR2* transcripts were evidently reduced in mitoTEMPO-treated shScr cells to levels similar to shMCU cells. On the hand, treatment of shMCU cells with antimycin A rescued mRNA expression of *TGFβ1, TGFβR1* and *TGFβR2* to levels comparable to shScr cells (Supplementary Fig. 9A). Previous studies have shown transcriptional regulation of TGFβ signalling by mROS via NF-κB and p38/JNK/ERK pathways [47, 48]. Control shScr cells treated with mitoTEMPO showed significant reduction in phosphorylated NF-κB (p-NF-κB) and phosphorylated p38 mitogen-activated protein kinases (p-p38 MAPK) protein levels with no change in total NF-κB and p38 MAPK expression, that was similar to shMCU cells (Supplementary Fig. 9B). Conversely, antimycin A treatment in shMCU cells rescued p-NF-κB and p-p38 MAPK expression to levels similar to that of control shScr cells (Supplementary Fig. 9B).

**Fig. 7:**
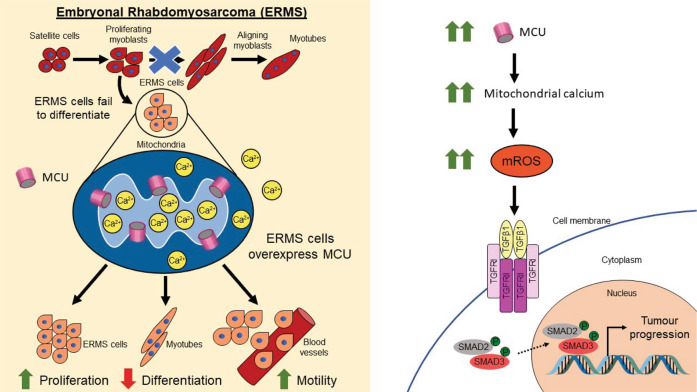
Graphical model of MCU function in ERMS. Mitochondrial calcium uniporter (MCU) is overexpressed in ERMS. Elevated mitochondrial calcium uptake due to MCU overexpression in the mitochondria promotes mROS production that activates TGFβ signalling and promotes tumour progression by increasing proliferation and motility with a decreased propensity to differentiate in vitro and in vivo.

Collectively, our data demonstrate that modulation of mROS production alters TGFβ signalling and oncogenic phenotypes in ERMS (Fig. 7).

## Discussion

The RAS pathway is frequently activated in ERMS and impacts redox balance [6, 29]. Consistently, ERMS cells are sensitive to drugs that elevate oxidative stress [29, 49]. Despite these correlations, the importance of mitochondrial Ca^2+^ homoeostasis has not been examined. Here we show that deregulated expression of the MCU complex impairs mitochondrial Ca^2+^ homoeostasis in ERMS cell lines. MCU knockdown caused a reduction in mitochondrial Ca^2+^ uptake and reduced mROS production. This inhibited the TGFβ signalling pathway and impaired proliferation and motility of tumour cells but promoted myogenic differentiation in vitro and in vivo.

Our finding that MCU positively regulates mitochondrial Ca^2+^ uptake is in concordance with previous studies on MCU knockout mice which show a lack of mitochondrial Ca^2+^ uptake [50, 51]. Moreover, an attenuation in mitochondrial Ca^2+^ uptake upon MCU knockdown has been reported in neurons [52], heart [53], liver [15] and pancreatic β cells [54]. The reduced OCR upon MCU knockdown is also in line with similar observations in myofibers of MCU knockout mice [55].

Mitochondria contribute to tumourigenesis and tumour progression in many ways that include the generation of ROS, accumulation of metabolites, and alterations in apoptosis [56]. Most of these processes are tightly regulated by Ca^2+^ ions. Since MCU and MICU1 regulate mitochondrial Ca^2+^ uptake and metabolism, deregulation in their expression leads to mitochondrial dysfunction. Indeed, studies have shown that increased or decreased MCU and MICU1 expression in different cancers contribute to tumourigenesis and metastasis in several ways [13, 19, 20]. In HCC as well as in breast cancer, MCU overexpression results in increased Ca^2+^ uptake and mROS generation, which play an important role in driving tumour progression and metastasis [21, 23]. Elevated mROS activates hypoxia-inducible factor 1-alpha (HIF1α), which promotes tumour progression [21]. Additionally, mROS has been reported to reduce superoxide dismutase 2 (SOD2) activity and promote ROS-dependent matrix metalloproteinase (MMP) activity, which promotes cell motility [23]. Interestingly, our RNA-Seq data identified a novel signalling pathway downstream of mROS production in ERMS. We show that TGFβ signalling is dampened in response to MCU knockdown. The interplay between ROS and TGFβ signalling pathway has been widely studied [34, 42, 57, 58] and both TGFβ ligands and receptors have been reported to be transcriptionally regulated by ROS via the p38 MAPK and NF-kB pathways [48]. TGFβ signalling is elevated in ERMS [59, 60]. Increasing or decreasing mROS modulated TGFβ signalling, demonstrating that elevated MCU-mediated mROS production is upstream of TGFβ signalling. Nevertheless, we note that integrins, which also regulate TGFβ signalling, are down regulated upon MCU knockdown. It is plausible that the reduced expression of these genes upon MCU knockdown also contributes to the reduction of TGFβ signalling. The TGFβ pathway has well-established roles in cell cycle progression and tumour invasion [33, 34]. In addition, TGFβ signalling potently represses myogenic differentiation [59, 61]. The impaired TGFβ signalling in shMCU cells correlates with the observed reduction in proliferation and cell motility and elevated myogenic differentiation in vitro and in vivo.

In some cancers, MCU overexpression protects cells from apoptosis and thus MCU silencing potentiates cell death [21, 62]. While ERMS cells overexpress MCU, we did not observe cell death in shMCU cells in vitro and in vivo. RNA-Seq analysis showed that the p53 pathway was also altered in response to MCU knockdown. The expression of several pro-apoptotic genes such as BH3-interacting domain death agonist (*BID*), tumour protein P53 (*TP53*), apoptotic protease activating factor 1 (*APAF1*) and phorbol-12-myristate-13-acetate-induced protein 1 (*PMAIP1*) [63] were reduced significantly upon MCU knockdown. The decreased expression of such pro-apoptotic genes may underlie the absence of apoptosis in shMCU cells. SNAI2 directly represses the pro-apoptotic gene *BIM/BCL2L11* expression in a p53-independent manner in RMS cell lines, and confers protection from ionising radiation [64]. As SNAI2 levels are also down regulated upon MCU knockdown, it would be interesting to determine the effect of radiation on these cells.

In addition to MCU overexpression, MICU1 is down regulated in ERMS cell lines. The regulatory mechanisms that underlie these changes in expression are unclear and need further investigation. As shMCU cells showed a modest impact on basal mitochondrial Ca^2+^ levels, it is likely that the down regulation of MICU1 in ERMS cell lines may contribute to the endogenous elevation of basal level of mitochondrial Ca^2+^ [16, 17].

Mitochondrial dysfunction is increasingly recognised to have central role in the development of several human diseases including cancer. Pharmacological interventions targeting mitochondria could become effective strategies for treating pathological conditions associated with mitochondrial dysfunction. However, the development of such therapeutic tools is hampered by the incomplete understanding of the molecular mechanisms underlying major mitochondrial functions. In this context, our study elucidates how high MCU expression is linked to tumour progression and a block in myogenic differentiation. Targeting the MCU-mROS-TGFβ axis could be a new unexplored therapeutic strategy in ERMS.

## Materials and methods

### Cell culture, transient and stable knockdown cells

Primary human skeletal muscle myoblasts (HSMM) were purchased from Zen-Bio, Inc. (NC, USA) and cultured in skeletal muscle cell growth medium (#SKM-M, Zen-Bio, USA). Cells were maintained at a confluency of no more than 70% and experiments were performed with cells under 7 passages. ERMS cell lines were a kind gift from Peter Houghton (Nationwide Children’s Hospital, Ohio, USA) and Rosella Rota (Bambino Gesu Children’s Hospital, Rome, Italy). RD cells were cultured in Dulbecco’s Modified Eagle Medium (DMEM) (Sigma-Aldrich, St. Louis, MO, USA) with 10% foetal bovine serum (FBS) (HyClone, Cytiva, U.S.) and 1% Penicillin-Streptomycin (HyClone, Cytiva, U.S.). RD18, JR1, RH36, RH30 and RH41 were cultured in RPMI 1640 with L-Glutamine (Thermo Fisher Scientific, Waltham, MA, USA) with 10% FBS and 1% Penicillin–Streptomycin. Mouse myoblasts (C2C12) were purchased from ATCC and cultured in DMEM with 20% FBS and 1% Penicillin–Streptomycin. The authenticity of all cell lines was confirmed by Short Tandem Repeats (STR) analyses (Axil Scientific Pte Ltd, Singapore).

For transient knockdown, cells were transfected with 50 nM human MCU-specific siRNA (ON-TARGETplus siRNA SMARTpool, Dharmacon, Lafayette, CO, USA) containing a pool of three to five 19-25 nucleotide siRNAs. Control cells were transfected with 50 nM scrambled siRNA (ON-TARGETplus, non-targeting pool, Dharmacon, Lafayette, CO, USA). Transfections were performed using Lipofectamine RNAiMax (Thermo Fisher scientific). Cells were analysed 48 h post-transfection for all assays.

For transient overexpression, cells were transiently transfected with 2.5 µg of pLYS5-MCU-Flag (#50054, Addgene). Control cells were transfected with 2.5 µg of pCMV-GFP (#11153, Addgene). Transfections were performed using Lipofectamine 3000 Transfection Reagent (Thermo Fisher Scientific). Cells were analysed 48 h post-transfection for all assays.

For generation of stable knockdown cell lines, HEK293FT cells were transfected with packaging plasmid pIP1 (5 µg) and pIP2 (5 µg), envelope plasmid pIP/VSV-G (5 µg) (ViraPower^TM^ Lentiviral Packaging Mix, Thermo Fisher Scientific) and 5 µg lentiviral expression constructs shRNA (pLKO.1 Mission shRNA DNA clone, Sigma-Aldrich Inc.) or shMCU (#SHCLNG-NM_138357 Mission shRNA, Sigma-Aldrich Inc.). 16 h post-transfection, the cell supernatant was replaced with basal DMEM medium. The supernatants were centrifuged, and the viral pellet was resuspended in DMEM medium. RD cells were transduced with shRNA control lentivirus particles or shMCU lentivirus particles with polybrene (8 µg/ml) (Sigma-Aldrich Inc.). Transduced cells were selected with 1 µg/ml puromycin (Sigma-Aldrich Inc.) for three days until all cells in control plates were dead.

### Mitochondrial calcium measurement

Cells were plated on glass bottom dishes and loaded with 5 µM Rhod-2 AM (Sigma-Aldrich Inc.) and 100 nM MitoTracker Green FM (Invitrogen) in extracellular medium as described previously [65, 66]. Cells were incubated for 50 min at 37 °C before washing with the same extracellular buffer containing 0.25% BSA at room temperature for 20 min. To measure mitochondrial Ca^2+^, the dishes were mounted on an on-stage incubator at 37 °C with 5% CO_2_ and imaged with confocal microscope with ×60 water objective lens. After 1 min of baseline recording, 100 μM histamine (Sigma-Aldrich Inc.) was added to induce mitochondrial Ca^2+^ uptake. Confocal images were recorded every 1 s at 561 nm excitation for another 4 min. The fluorescence intensities of the images were analysed and quantified with Image J (NIH). Mitochondrial Ca^2+^ changes were quantified by plotting relative fluorescence intensity of the images for a duration of 5 min. Basal mitochondrial Ca^2+^ was quantified by measuring relative fluorescence intensity during the first 1 min of baseline recording. Maximal mitochondrial Ca^2+^ uptake was quantified by the difference between maximal fluorescence intensity and basal fluorescence intensity.

### Reactive oxygen species

Cellular ROS and mitochondrial superoxide were detected using fluorescence probe CM-H_2_DCFDA (Invitrogen; Thermo Fisher Scientific, Inc., USA) and MitoSOX Red (Invitrogen; Thermo Fisher Scientific, Inc., USA) respectively. Cells were trypsinised and loaded with 5 μM CM-H_2_DCFDA or 5 μM MitoSOX Red for 20 min at 37 °C, respectively. Fluorescence intensity was analysed using flow cytometry. A minimum of 100,000 events per sample were collected and the data was analysed using CytExpert software (Beckman Coulter, Inc.). To modulate mROS levels, RD cells were treated with 200 nM of mitoTEMPO (mT), whereas shMCU cells were treated with 500 nM antimycin A (AA) for 48 h. DMSO was used as a control.

To measure mitochondrial hydrogen peroxide, cells were transfected with pC1-HyPerRed-mito (Addgene plasmid #60247). Forty-eight hours post transfection, cells were imaged with confocal microscope with ×60 water objective lens. HyperRed-mito fluorescence intensity was analysed by Image J software (NIH).

### ATP measurement

ATP production was measured with the ATPlite Luminescence Assay System (PerkinElmer) according to the manufacturer’s instructions.

### Oxygen consumption rate measurement

Oxygen consumption rate (OCR) was measured with a XF24 extracellular analyser (Seahorse Bioscience) and XF Cell Mito Stress Test Kit (Seahorse Bioscience). Cells were seeded at 50,000 cells/well (~80–90% confluent when assayed) in a 24-well Agilent Seahorse XF Cell Culture Microplate (Seahorse Bioscience) and incubated overnight at 37 °C. Prior to the assay, growth media was replaced with XF DMEM medium, pH 7.4 (Seahorse Bioscience) supplemented with 1 mM sodium pyruvate (Sigma-Aldrich, St. Louis, MO, USA) and 10 mM glucose (Sigma-Aldrich, St. Louis, MO, USA). Cells were then incubated for 45 min to 1 h in 37 °C without CO_2_ to prevent acidification of medium. After loading the plate into the machine, basal respiration rate was measured before cells were exposed sequentially to oligomycin (1 μM), carbonyl cyanide p-trifluoromethoxyphenylhydrazone (FCCP; 1 μM) and rotenone + antimycin A (500 nM each). After each injection, OCR was measured for 5 min, the medium was mixed and again measured for another 5 min. After the experiment, protein concentration was determined by lysing samples in each well and performing Bradford analysis (Bio-Rad). Maximum respiration rate was quantified by maximal OCR after adding FCCP. ATP-linked respiration was quantified by the decrease in OCR upon injection of the ATP synthase inhibitor oligomycin.

### Reporter assays

TGFβ reporter assay was analysed as described [67]. Briefly, shScr and shMCU cells were transfected with 200 ng of 3TP-Lux reporter in 24-well plates. 5 ng of Renilla reporter was co-transfected as an internal normalisation control. Transfection was carried out in triplicates using Lipofectamine 3000 Transfection Reagent (Thermo Fisher Scientific). Reporter activity was analysed with the Dual-Luciferase Reporter Assay System (Promega). Luminescence was analysed with Varioskan plate reader using the SkanIT software.

### Western blot analysis

Whole-cell and tumour lysates were isolated using RIPA buffer supplemented with protease inhibitors (Complete Mini, Sigma-Aldrich Inc.) and phosphatase inhibitors including sodium pyrophosphate, β-glycerophosphate, sodium fluoride and sodium orthovanadate (Sigma-Aldrich). The following primary antibodies were used: anti-MCU (#D2Z3B 1:1000, Cell Signaling), anti-MICU1 (#HPA037479 1:1000, Sigma-Aldrich), anti-MICU2 (#ab101465 1:1000, Abcam), anti-phospho-SMAD3 (#C25A9, 1:1000, Cell Signaling), anti-SMAD3 (#9513, 1:1000, Cell Signaling), anti-MYOG (#sc-12732, 1:500, Santa-Cruz), anti-MHC (#sc-32732, 1:250, Santa-Cruz) anti-HSP60 (#611563, BD Biosciences), anti-phospho-NF-κB (#3037, 1:1000, Cell Signaling), anti-NF-κB (#ab52175, 1:500, Abcam), anti-phospho-p38 MAPK (#9211, 1:1000, Cell Signaling), anti-p38 MAPK (#9212, 1:1000, Cell Signaling), and anti-β-actin (#A2228, 1:10,000, Sigma-Aldrich). Appropriate secondary antibodies (IgG-Fc Specific-Peroxidase) of mouse or rabbit origin (Sigma-Aldrich) were used.

### RNA sequencing (RNA-Seq) and quantitative real-time polymerase chain reaction (qPCR)

For RNA-Seq analysis, RNA was isolated from control (siScr) and siMCU cells in triplicates using Trizol. RNA purity and integrity were assessed with Nanodrop, agarose gel electrophoresis and Agilent 2100. Raw image data file from Illumina (HiSeq PE150) was transformed to Sequenced Reads by CASAVA base recognition and stored in FASTQ(fq) format. Raw reads were filtered in order to achieve clean reads using the following filtering conditions: reads without adaptors, reads containing number of base that cannot be determined below 10% and at least 50% bases of the reads having Qscore denoting Quality value ≤5. For mapping of the reads, STAR software was used. 1 M base was used as the sliding window for distribution of the mapped reads. For the analysis of differentially expressed genes, Gene Ontology (GO) and Kyoto Encyclopaedia of Genes and Genomes (KEGG) analysis were done with corrected *p* value <0.05 as significant enrichment.

For qPCR analysis, total RNA was extracted using Trizol (Thermo Fisher Scientific) and quantified using Nanodrop. Messenger RNA (mRNA) was converted to a single-stranded complementary DNA (cDNA) using iScript cDNA Synthesis Kit (Bio-Rad). qPCR was performed using Lightcycler 480 SYBR Green 1 Master Kit (Roche). PCR amplification was performed as follows: 95 °C 5 min, followed by 95 °C for 10 s, annealing at 60 °C for 10 s, and 45 cycles at 72 °C for 10 s. Light Cycler 480 software (version 1.3.0.0705) was used for analysis. CT values of samples were normalised to internal control GAPDH to obtain delta CT (ΔCT). Relative expression was calculated by 2^−ΔCT^ equation. qPCR was done using technical triplicates and at least three independent biological replicates were done for each analysis. Representative data is shown. Primer sequences can be found in Supplementary Table 1. RNA-Seq data has been deposited in GEO under the accession number GSE173200.

### Proliferation and differentiation assays

Proliferation and differentiation were analysed as described [68, 69]. Briefly, proliferation was measured using 5-bromo-2’-deoxy-uridine (BrdU) labelling (Roche, Basel, Switzerland). Cells were pulsed with 10 μM BrdU, fixed and incubated with anti-BrdU antibody (1:100) followed by anti-mouse Ig-fluorescein antibody (1:200) and mounted onto a glass slide using DAPI (Vectashield, Vector Laboratories, CA, USA). Images were captured using fluorescence microscope BX53 (Olympus Corporation, Shinjuku, Tokyo, Japan) at ×40 magnification.

For differentiation, cells were cultured in differentiation media consisting of either basal DMEM or RPMI 1640 with 2% Horse Serum (HyClone, Cytiva, U.S.) for 2–5 days. Cells were incubated with anti-MHC primary antibody (MHC; R&D Systems, Minneapolis, MN, USA) (1:400) followed by secondary goat anti-Mouse IgG (H + L) Highly Cross-Adsorbed Secondary Antibody, Alexa Fluor 568 (Thermo Fisher scientific). Coverslips were mounted with DAPI (Vectashield, Vector Laboratories, CA, USA) and imaged with BX53 microscope (Olympus Corporation) at ×40 magnification.

### Migration and invasion assay

Migratory and invasive capacity were assessed as described [68, 69] with Boyden chamber (Greiner Bio-One). Briefly, cells were serum deprived for at least 12 h and seeded at a density of 50,000 cell per well in serum-free media. In all, 10% FBS-containing media was added to the lower chamber. The inserts were stained with crystal violet after 24 h and imaged at ×10 magnification. The invasive capacity of the cells was determined similarly using inserts coated with matrigel (Bio Lab) and cells were seeded at a density of 70,000 cells per insert.

### Mouse xenograft experiments

Six-week-old C.Cg/AnNTac-Foxn1^nu^NE9 female BALB/c nude mice (InVivos, Singapore) were injected subcutaneously in the right flank with either shScr or shMCU RD cells (10^6^ cells per mice). 7 mice were used randomly in each group. The number of mice per group was determined using power analysis assuming 5% significance level and 80% statistical power with 10% attrition rate. Tumour onset and growth were monitored every alternate day. Tumour diameter was measured, and volume was calculated using the following formula: *V* = (*L* × *W* × *W*)/2, where *V* = tumour volume, *L* = tumour length, *W* = tumour width. Resected tumours were used to prepare tumour lysates for western blot analysis or fixed with formalin for histopathological analysis. No blinding was done for the analysis. All animal procedures were approved by the Institutional Animal Care and Use Committee under the protocol number R19-0890.

### Immunohistochemistry (IHC)

Paraffin sections of 6 archival primary ERMS tumours from KK Women’s and Children Hospital in Singapore were analysed by IHC using anti-MCU antibody (1:50, Sigma-Aldrich). Following Institutional Review Board approval (CIRB 2014/20179), specimens were obtained from patients at KK Women’s and Children Hospital who were recruited prospectively, with written parental consent and child assent obtained. TMA (SO2082b), comprising of 27 ERMS tumour specimens, 24 ARMS tumour specimens and 8 striated muscle tissue, was purchased from US Biomax, Inc. and analysed by IHC using anti-MCU antibody (1:50, Sigma-Aldrich) following the manufacturer’s protocol. Paraffin sections from mouse xenografts were stained with haematoxylin and eosin and analysed by IHC as described [68, 69]. Sections were incubated overnight at 4 °C with anti-MCU (1:50, Sigma-Aldrich), anti-Ki67 (1:100, Santa Cruz Biotechnology), anti-MCAM (1:200, Proteintech), anti-SNAI2 (1:100, Proteintech), anti-active caspase 3 (1:200, Cell Signaling), anti-MHC (1:200, Santa Cruz Biotechnology) antibodies using Dako REAL EnVIsion-HPR, Rabbit-Mouse kit (Dako, Denmark). Sections were counterstained with haematoxylin (Sigma-Aldrich). Slides were dehydrated and mounted using DPX (Sigma-Aldrich) and imaged using BX53 Olympus microscope at ×40 magnification.

### Statistical analysis

For statistical analysis, two-tailed non-parametric unpaired *t* test was used to evaluate the significance between data sets with the use of GraphPad prism 9.0 software. For animal xenograft experiment and rescue experiments, one-way analysis of variance test (ANOVA) with appropriate correction was performed with the GraphPad prism 9.0 software. Each experiment was performed at least thrice as independent biological replicates. Each independent experiment had three technical replicates with the exception of migration and invasion assay which had two technical replicates each. All technical replicates were plotted on the scatter plots. Standard error of mean was calculated for all data sets and a *p* value <0.05 was considered statistically significant.

## Supplementary information

Supplementary data

Checklist

Original Data File

Original Data File

Original Data File

Original Data File

## Data Availability

The RNA-seq data have been deposited in GEO under the accession number GSE173200 and can be viewed with the token: gbexwwkifjgrdip.
