# Supplementary material for: Mitochondrial calcium uptake regulates tumour progression in embryonal rhabdomyosarcoma
Source: Cell Death Dis. 2022 Apr 30;13(4):419. doi: 10.1038/s41419-022-04835-4 (PMC9056521; doi:10.1038/s41419-022-04835-4)
Supplement: Supplementary file 1 — Supplementary data [file 41419_2022_4835_MOESM1_ESM.pdf]

Supplementary Figure Legends

Supplementary Fig. 1: MCU is overexpressed in ERMS patient tumours

TMA consisting of 8 normal skeletal muscle samples, 24 ARMS and 27 ERMS patient tumour specimens was analysed by IHC using anti-MCU antibody. Images were taken at 40X magnification. Inset shows 3X zoomed in image. Scale bar: 50μm.

Supplementary Fig. 2: MCU regulates mitochondrial functions in JR1 cell line

A Western blot analysis showed significant downregulation of MCU expression in transient MCU knockdown JR1 cells with no change in HSP60 expression. β-actin was used as loading control. The western blot is representative of 3 independent experiments. **B** Basal and maximal mitochondrial Ca^{2+} uptake upon induction with 100μM histamine using Rhod2-AM staining is shown in control and siMCU cells (n=3). Values correspond to the average ± SEM. **C** MitoSOX Red staining of siMCU in comparison to siScr is shown (n=3). Values correspond to average ± SEM. **D** OCR was measured in siMCU cells compared to siScr cells. O: Oligomycin, FCCP: Carbonyl cyanide-4-(trifluoromethoxy)-phenylhydrazone, AA+R: Antimycin A and Rotenone were added accordingly. Values correspond to average ± SEM (n=3). **E** Basal and maximal respiration rate, along with ATP-linked respiration in siScr and siMCU cells were shown. Values correspond to average ± SEM (n=3). Two-tailed non-parametric unpaired t-test was performed for statistical analysis. ns = not significant, *** $p \leq 0.001$, **** $p \leq 0.0001$.

Supplementary Fig. 3. MCU regulates mitochondrial Ca^{2+}, mROS, TGFβ signalling and proliferation in myoblasts

A Western blot analysis showed significant upregulation of MCU expression in transient MCU overexpressing C2C12 cells. The western blot is representative of 3 independent experiments. **B** Basal and maximal mitochondrial Ca^{2+} uptake upon induction with 100μM histamine using Rhod2-AM staining is shown in control (pCMV) and MCU overexpressing (pMCU) cells (n=3). Values correspond to the average ± SEM. **C** MitoSOX Red staining of pMCU in comparison

to pCMV is shown (n=3). Values correspond to average ± SEM. **D** pCMV and pMCU cells were transfected with the p3TP-Lux and analysed for luciferase activity 48hr later. The values correspond to average ± SEM (n=2). **E** BrdU assay to examine proliferation in pCMV and pMCU cells. $BrdU^+$ cells were analysed by immunofluorescence (n=5). Images are representative of 5 independent experiments. Scale bar: 50µm. The scatter plot shows the percentage of $BrdU^+$ cells in pMCU cells relative to control cells. The values correspond to average ± SEM. Two-tailed non-parametric unpaired t-test was performed for statistical analysis. ns = not significant, **** $p \leq 0.0001$.

Supplementary Fig. 4: MCU knockdown inhibits proliferation

A. Proliferation was analysed in siScr and siMCU RD cells using BrdU assay. Cells were analysed by immunofluorescence (n=3). Images are representative of 3 independent experiments. Scale bar: 50µm. The scatter plot shows the percentage of $BrdU^+$ cells in siMCU cells relative to controls. The values correspond to average ± SEM. **B**. Proliferation was analysed in siScr and siMCU RD18 cells (n=3). Scale bar: 50µm. The scatter plot shows the percentage of $BrdU^+$ cells. The values correspond to average ± SEM. **C**. Proliferation was analysed in siScr and siMCU JR1 cells (n=3). Scale bar: 50µm. The scatter plot shows the percentage of $BrdU^+$ cells. The values correspond to average ± SEM. **D**. Proliferation was analysed in siScr and siMCU RH36 cells (n=3). Scale bar: 50µm. The scatter plot shows the percentage of $BrdU^+$ cells. The values correspond to average ± SEM. Two-tailed non-parametric unpaired t-test was performed for statistical analysis. *** $p \leq 0.001$, **** $p \leq 0.0001$.

Supplementary Fig. 5: MCU knockdown promotes differentiation

A Control siScr and siMCU RD cells were cultured for 5 days in differentiation medium. Cells were analysed by immunofluorescence using anti-MHC antibody. Nuclei were stained with DAPI. Representative images of 3 independent experiments are shown. Scale bar: 50µm. The scatter plot shows the percentage of MHC^+ cells. The values correspond to average ± SEM. **B** Control siScr and siMCU RD18 cells analyzed after 5 days in differentiation medium using

anti-MHC antibody (n=3). Scale bar: 50μm. The scatter plot shows the percentage of MHC^+ cells. The values correspond to average ± SEM. **C** Control siScr and siMCU JR1 cells were differentiated for 5 days in differentiation medium. (n=3). Scale bar: 50μm. The scatter plot shows the percentage of MHC^+ cells. The values correspond to average ± SEM. **D** Control siScr and siMCU RH36 cells were differentiated for 5 days in differentiation medium. (n=3). Scale bar: 50μm. The scatter plot shows the percentage of MHC^+ cells. The values correspond to average ± SEM. Two-tailed non-parametric unpaired t-test was performed for statistical analysis. **** $p \leq 0.0001$.

Supplementary Fig: 6. MCU knockdown inhibits motility

A. Migration of control RD and siMCU cells was observed after 24hr by crystal violet staining. Images are representative of 4 independent experiments. Scale bar: 200μm. The relative percentage of migrated cells were quantified in the scatter plot and the values correspond to average ± SEM. **B** Migration of control and siMCU RD18 cells was analysed after 40hr by crystal violet staining (n=4). The scatter plot shows the percentage of migrated cells and the values correspond to average ± SEM. **C** Migration of control and siMCU JR1 cells after 24hr was assessed by crystal violet staining (n=4). Scale bar: 200μm. The relative percentage of migrated cells were quantified in the scatter plot and the values correspond to average ± SEM. **D** Migration of control and siMCU RH36 cells after 24hr was assessed by crystal violet staining (n=3). Scale bar: 200μm. The relative percentage of migrated cells were quantified in the scatter plot and the values correspond to average ± SEM. Two-tailed non-parametric unpaired t-test was performed for statistical analysis. **** $p \leq 0.0001$.

Supplementary Fig. 7: MCU knockdown does not affect oncogenic phenotypes in RH30 cells. Minimal TGFβ pathway activation in ARMS cells

A Western blot analysis showed significant downregulation of MCU expression in transient MCU knockdown RH30 cells. β-actin was used as loading control. The western blot is representative of 3 independent experiments. **B-C** BrdU assay to examine proliferation in siScr

and siMCU cells. BrdU$^+$ cells were analysed by immunofluorescence (n=3). Images are representative of 3 independent experiments. Scale bar: 50μm. The scatter plot shows the percentage of BrdU$^+$ cells in siMCU cells relative to controls. The values correspond to average ± SEM. **D** Control siScr and siMCU cells were cultured for 3 days in differentiation medium and analysed by immunofluorescence using anti-MHC antibody. Nuclei were stained with DAPI. Representative images of 3 independent experiments are shown. Scale bar: 50μm. The scatter plot shows the percentage of MHC$^+$ cells in siMCU cells relative to controls. The values correspond to average ± SEM. **E** MYOG level was analysed in control and siMCU cells by western blot analysis at Day 2 in differentiation medium. The western blot is representative of 3 independent experiments. **F-G** Boyden chamber migration assay of control and siMCU cells. Migrated cells were observed after 24hr using crystal violet staining. Images are representative of 3 independent experiments. Scale bar: 200μm. The relative percentage of migrated cells were quantified in the scatter plot and the values correspond to average ± SEM. **F** Western blot analysis of TGFβ pathway activation in HSMM, RD, RH30 and RH41 cells using p-SMAD3 and SMAD3 antibodies. β-actin was used as loading control. A representative western blot from 3 independent experiments is shown. Two-tailed non-parametric unpaired t-test was performed for statistical analysis. ns = not significant.

Supplementary Fig. 8: RNA sequencing analysis and downstream transcriptional targets of TGFβ signalling

A-B Selected gene lists of up and down regulated genes in the top 5 significantly enriched GO analysis (**A**) and KEGG pathway analysis (**B**). **C** qPCR validation of myostatin (*MSTN*) and Hairy/enhancer-of-split related with YRPW motif protein 2 (*HEY2)* in control and siMCU cells. The values correspond to average ± SEM (n=3). **D** qPCR validation of top 3 differentially regulated integrins, *ITGB3, ITGA2* and *ITGA7* in control shScr and shMCU cells. The values correspond to average ± SEM (n=3). **E** qPCR validation of latent transforming growth factor beta binding protein 2 (*LTBP2), LTBP4,* matrix metallopeptidase 16 (*MMP16),* Metalloproteinase inhibitor 3 *(TIMP3)* and serpin family E member 1 (*SERPINE1)* in control

shScr and shMCU cells. The values correspond to average ± SEM (n=3). Two-tailed non-parametric unpaired t-test was performed for statistical analysis. *** $p \leq 0.001$, **** $p \leq 0.0001$.

Supplementary Fig. 9: mROS regulated TGFβ signalling through NFκB/p38 MAPK pathway

A shScr and shMCU RD cells were treated with DMSO, mitoTEMPO (mT) or antimycin A (AA) for 48hr. *TGFβ1, TGFβR1* and *TGFβR2* mRNA expression were significantly reduced in mitoTEMPO treated shScr cells similar to shMCU cells. Treatment of shMCU cells with antimycin A rescued mRNA expression of *TGFβ1, TGFβR1* and *TGFβR2* to comparable levels as shScr cells. The values correspond to average ± SEM (n=3). **B** p-NFκB, NFκB, p-p38 MAPK and p38 MAPK levels were examined by western blot analysis in shScr and shMCU cells treated with mT and AA. Representative images of 3 independent experiments are shown. One-way ANOVA test with appropriate correction was performed for statistical analysis. ns = not significant, * $p \leq 0.05$, ** $p \leq 0.01$, **** $p \leq 0.0001$.

Supplementary Table Legend

Supplementary Table 1: Primer sequences used for qPCR.

Supplementary Fig. 1

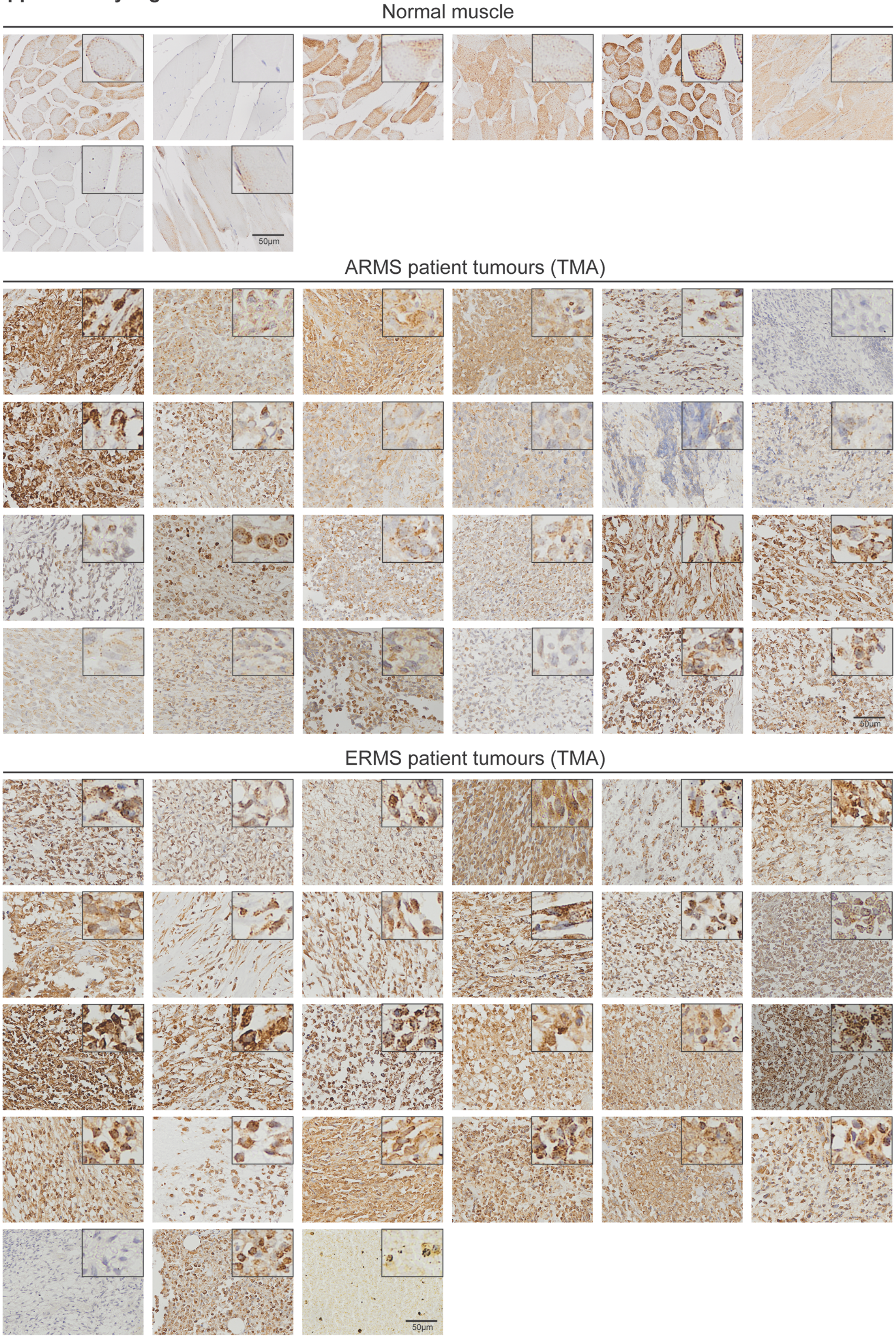

Supplementary Fig. 2

Cell line: JR1

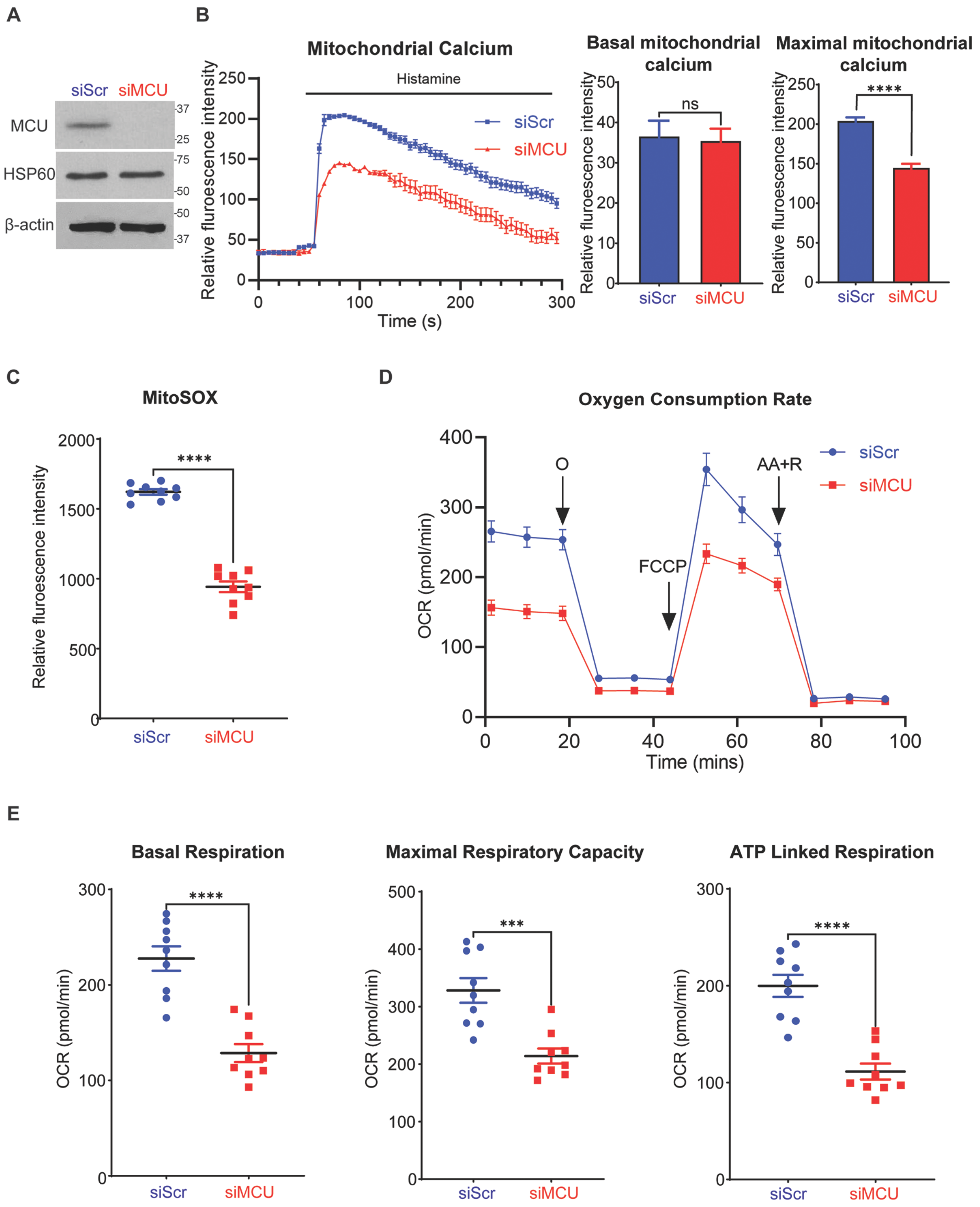

Supplementary Fig. 3

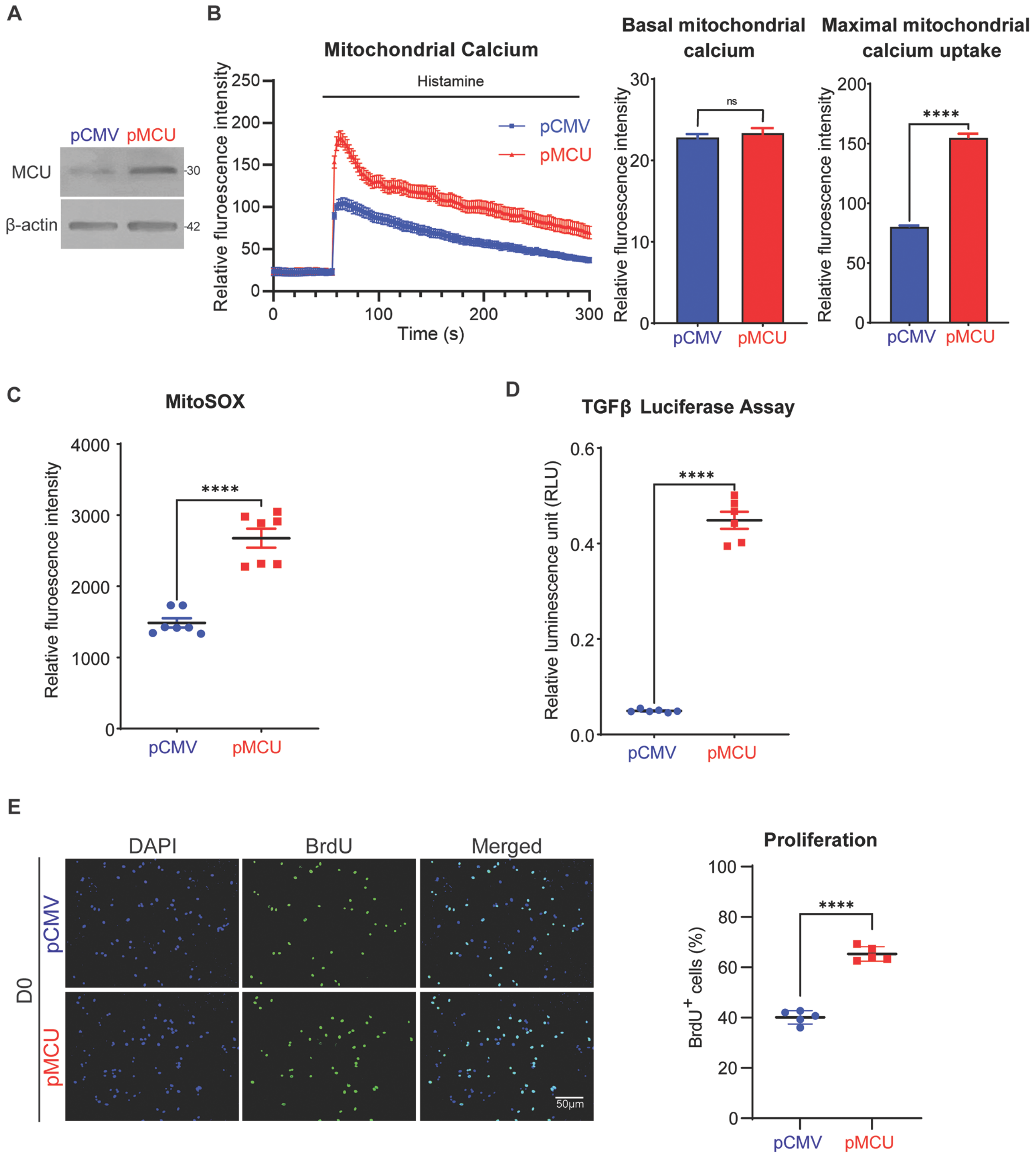

Supplementary Fig. 4

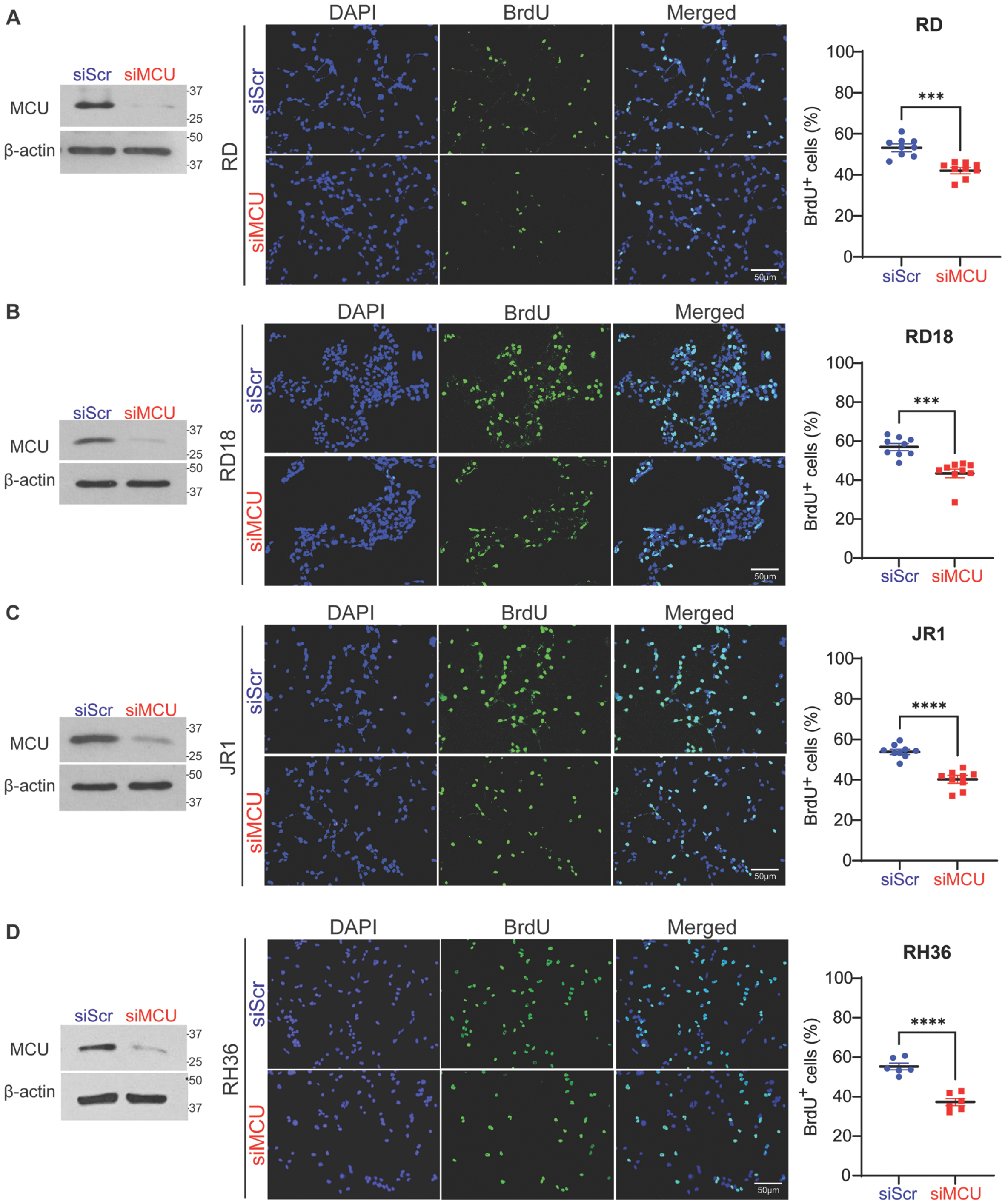

Supplementary Fig. 5

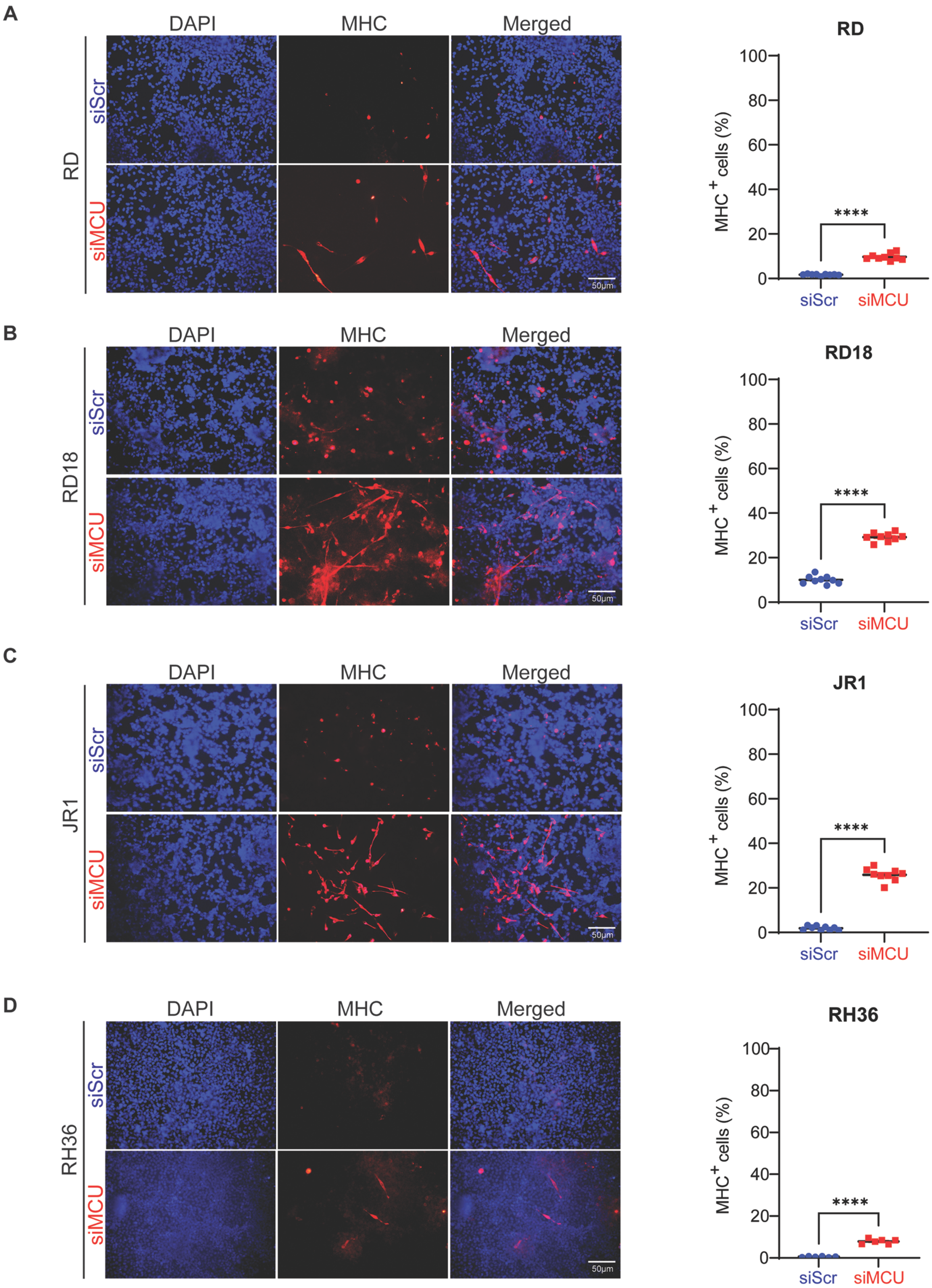

Supplementary Fig. 6

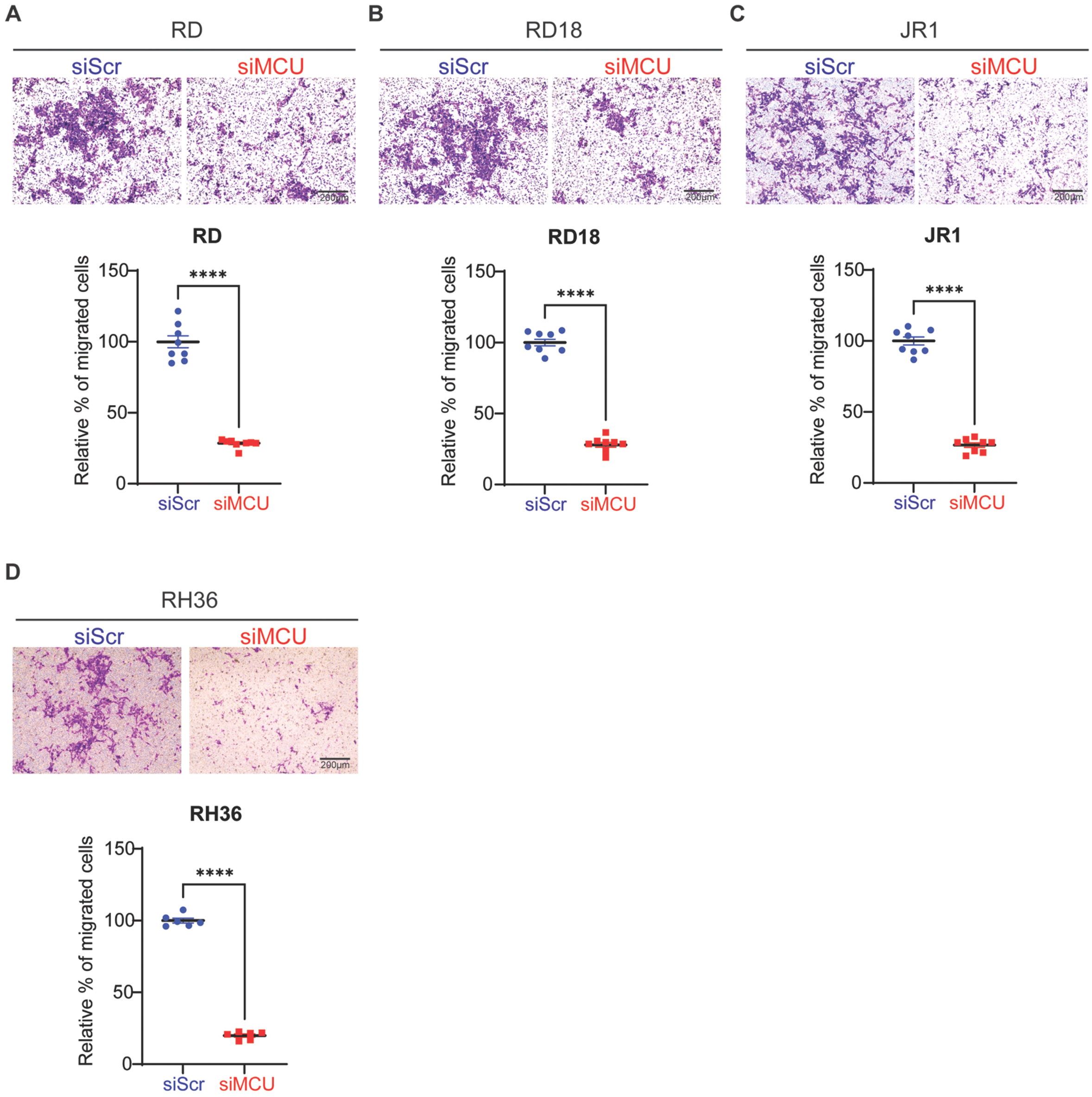

Supplementary Fig. 7
Cell line: RH30

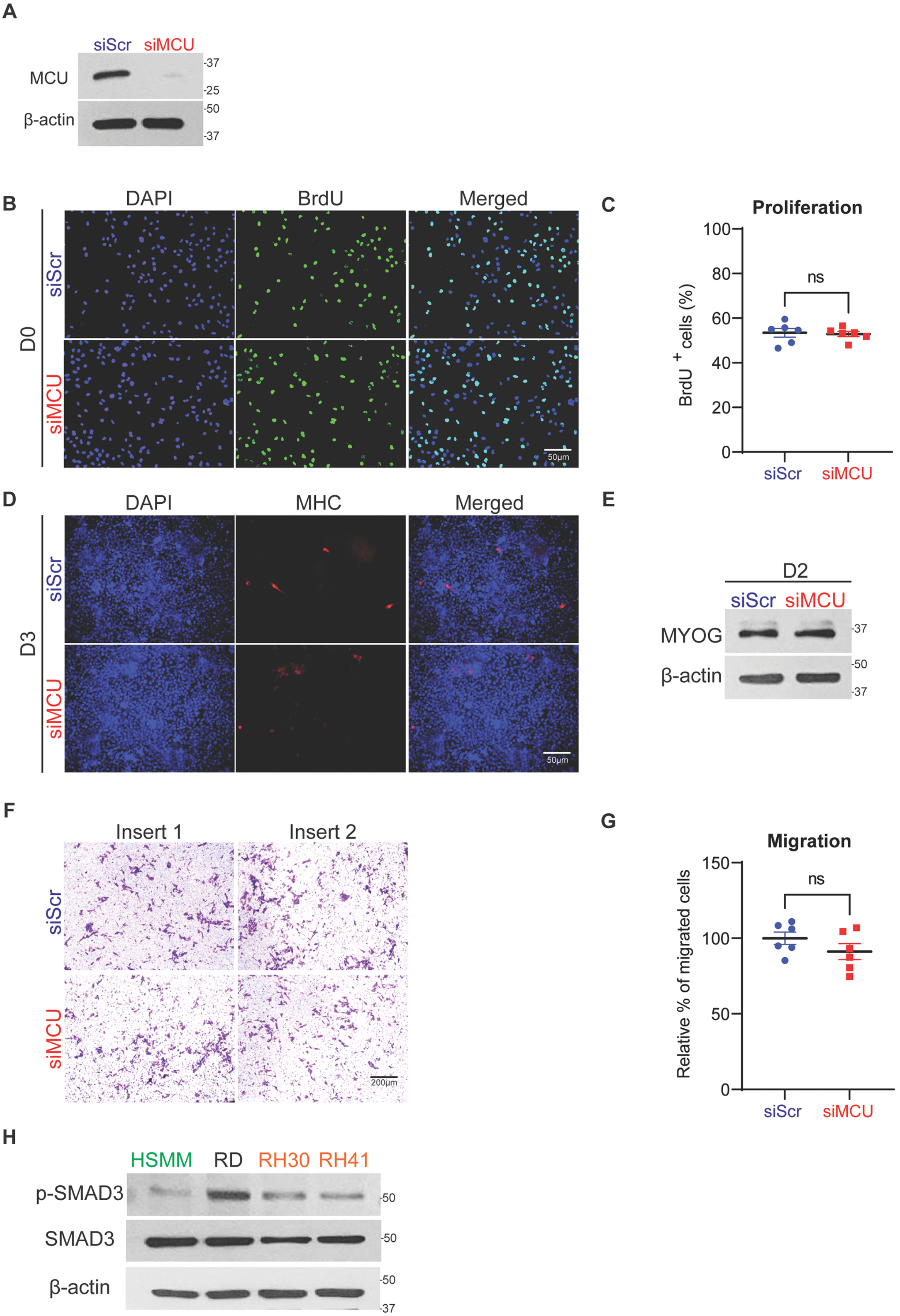

Supplementary Fig. 8

A

GO	Regulation	Gene	Fold Change	p-adjusted
Skeletal system development	Upregulated	OSR1	1.604707108	9.6811E-07
	Downregulated	TGFBR1	1.726631244	2.7398E-40
	Downregulated	CD44	1.579962153	1.1202E-36
	Downregulated	IGF2	1.346645392	8.7989E-11
	Downregulated	MMP16	1.292397621	0.0064157
Posttranscriptional regulation of gene expression	Upregulated	WTIP	1.438225605	0.00066918
	Upregulated	FOXO3	1.348382679	1.4375E-08
	Downregulated	PSMB5	1.441459198	1.3374E-22
	Downregulated	UHMK1	1.347616502	5.7462E-14
	Downregulated	RPS6KB1	1.308115564	7.8142E-06
Focal adhesion	Downregulated	ITGB3	1.821301794	2.599E-11
	Downregulated	ITGA3	1.733947238	7.7612E-18
	Downregulated	CD44	1.579962153	1.1202E-36
	Downregulated	PLAUR	1.465842399	7.7792E-18
	Downregulated	MCAM	1.303915157	3.3454E-08
Muscle organ development	Upregulated	ALX4	1.417393168	1.127E-09
	Downregulated	TGFBR1	1.726631244	2.7398E-40
	Downregulated	MSTN	1.618502955	1.1377E-08
	Downregulated	HEY2	1.321979679	0.0034364
	Downregulated	TGFBR2	1.276452631	1.1214E-10
G1/S transition of mitotic cell cycle	Upregulated	CTDSP2	1.310902148	1.5814E-15
	Upregulated	FHL1	1.301386957	3.3303E-07
	Downregulated	PLRG1	1.363098672	6.9134E-10
	Downregulated	UBB	1.292926264	7.8137E-11
	Downregulated	MCM3	1.274136638	3.7249E-11

B

KEGG pathway	Regulation	Gene	Fold Change	p-adjusted
Cell cycle	Upregulated	CDKN1C	1.273571538	0.012849
	Upregulated	GADD45B	1.263346164	0.0019176
	Downregulated	MCM3	1.274136638	3.7249E-11
	Downregulated	SKP2	1.270089356	4.2152E-10
	Downregulated	CDC20	1.187921909	9.2334E-06
Ubiquitin mediated proteolysis	Upregulated	SOCS1	1.199913161	0.04024
	Downregulated	UBE3C	1.406909959	5.2281E-24
	Downregulated	UBE2S	1.278985581	1.5571E-08
	Downregulated	SKP2	1.270089356	4.2152E-10
	Downregulated	UBE2D1	1.245823766	0.00080786
p53 signaling pathway	Downregulated	GADD45A	1.417383343	7.3239E-17
	Downregulated	PMAIP1	1.370041885	6.1836E-16
	Downregulated	APAF1	1.258704824	3.7482E-06
	Downregulated	BID	1.154733555	0.011454
	Downregulated	TP53	1.131102757	0.028018
TGF-beta signaling pathway	Downregulated	TGFBR1	1.726631244	2.7398E-40
	Downregulated	THBS2	1.414752807	5.5299E-21
	Downregulated	TGFBR2	1.276452631	1.1214E-10
	Downregulated	TGFB1	1.226179338	0.046371
	Downregulated	MAPK1	1.102508669	0.029257
ECM-receptor interaction	Downregulated	ITGB3	1.821301794	2.599E-11
	Downregulated	ITGA3	1.733947238	7.7612E-18
	Downregulated	CD44	1.579962153	1.1202E-36
	Downregulated	THBS2	1.414752807	5.5299E-21
	Downregulated	ITGA7	1.281692343	4.9904E-06

C

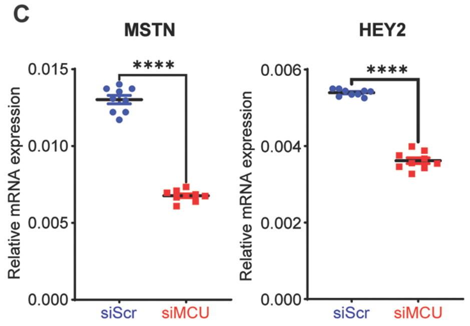

D

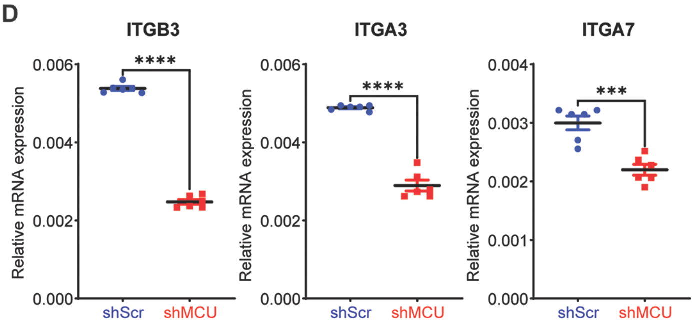

E

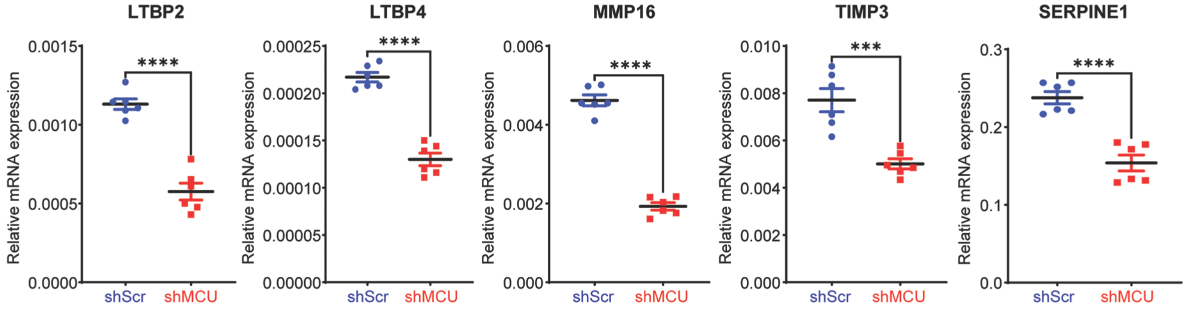

Supplementary Fig. 9

A

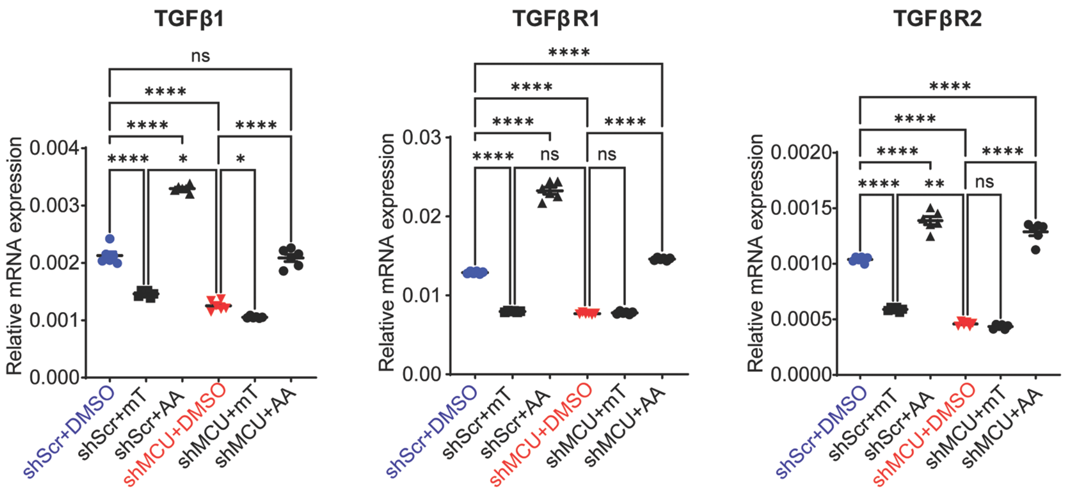

B

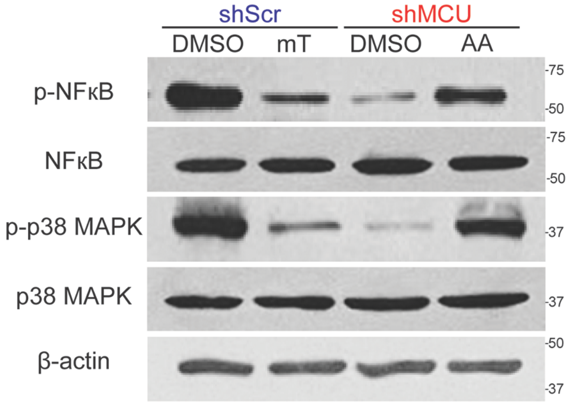

Supplementary Table S1: Primer sequences used for q-PCR

Gene	Forward sequence	Reverse sequence
MCU	GTCAGTTCACACTCAAGCCTAT	TTGAAGCAGCAACGCGAACA
TGFβ1	GGAAATTGAGGGCTTTCGCC	CCGGTAGTGAACCCGTTGAT
TGFβR1	GAACTTCCAACTACTGGCCCT	AATGACAGCTGCCAGTTCCA
TGFβR2	GCAGCGCTGAGTTGAAGTTG	GAGGGAAGCTGCACAGGAG
MSTN	CAACGGTGCTAATACGATAGGC	GAGGTGTAGGAAAATGCACCTG
IGF2	GCTGTTCGGTTTGCGACAC	GGGATTCCCATTGGTGTCTGG
FGFR4	ACCCCACGCCCACCAT	TGCGGTTCTCCCCATGAA
HEY2	TTGAAGATGCTTCAGGCAACAGGG	TCAGGTACCGCGCAACTTCTGTTA
WNT2B	AAAAGGGGCCAGGAGGATTC	GCTGGCTCTTGCTTGCTTAC
WNT5A	GCCAGTATCAATTCCGACATCG	TCACCGCGTATGTGAAGGC
LTBP2	CGGTGATTGAGAATGGCCAG	GTATTCACACACTCCGCGTC
LTBP4	CGACATGCCAGACTTTGAGG	ACCAGCATAGCTTCCACCTT
MMP16	AGCACTGGAAGACGGTTGG	CTCCGTTCCGCAGACTGTA
TIMP3	TGCTCTCTGTCTCTTTTTTCAGCTT	CTACAGTGTGTTGTCTGCTGCTTTT
SERPINE1	TCGAGGTGAACGAGAGTGGCA	AAGGACTGTTCCTGTGGGGTTGT
ITGA3	GCCTGCCAAGCTAATGAGAC	ACCTGAAGGTCCCTTGTGTG
ITGA7	GCTGTGAAGTCCCTGGAAGTGATT	GCATCTCGGAGCATCAAGTTCTT
ITGB3	CATCACCATCCACGACCGAA	GTGCCCCGGTACGTGATATT
