# Supplementary material for: Mitochondrial calcium uptake regulates tumour progression in embryonal rhabdomyosarcoma
Source: Cell Death Dis. 2022 Apr 30;13(4):419. doi: 10.1038/s41419-022-04835-4 (PMC9056521; doi:10.1038/s41419-022-04835-4)

Figure 1D

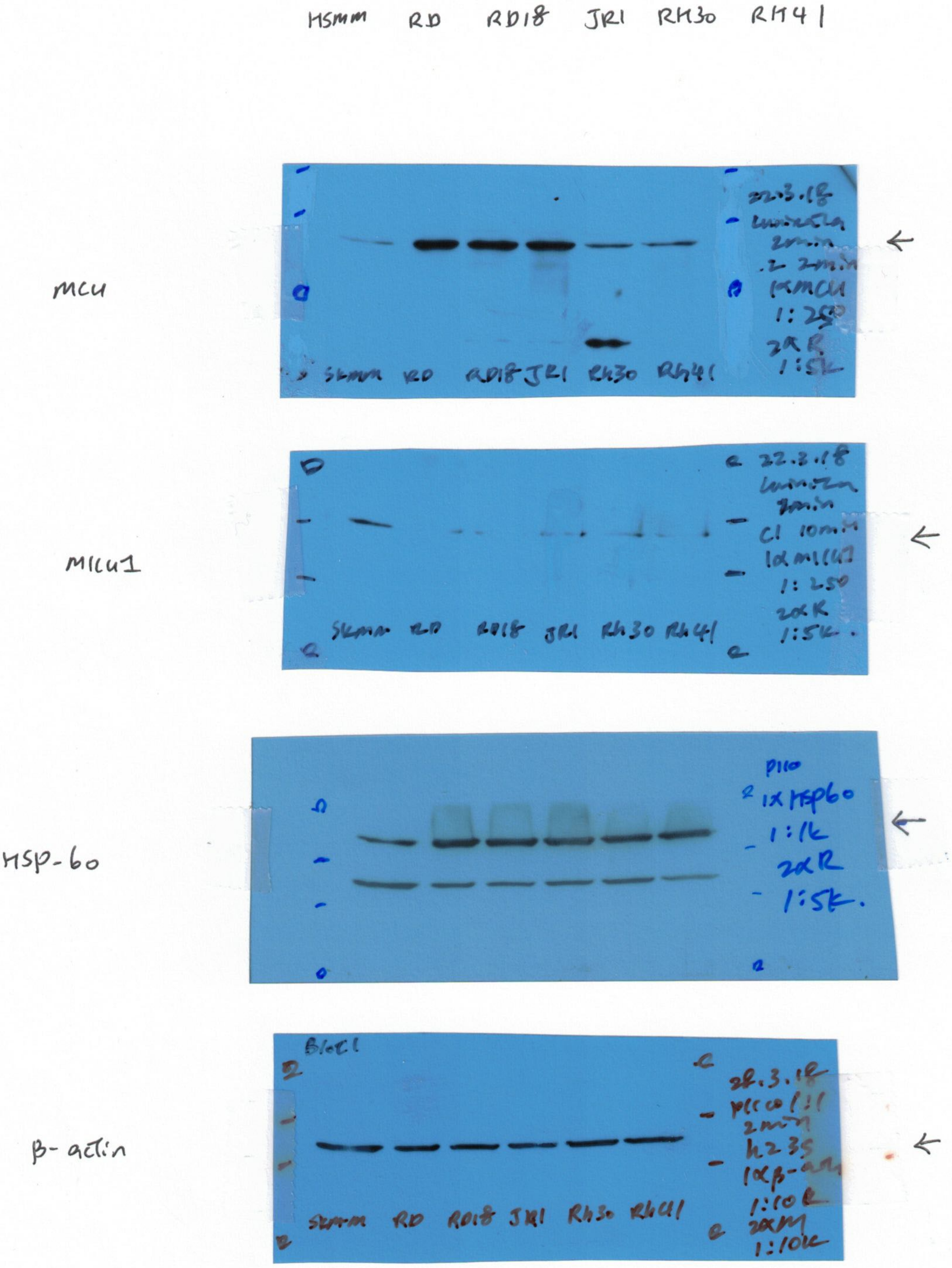
HSMM RD RD18 JR1 RH30 RH41
MCU
MICU1
HSP-60
β-actin
22.3.18
1:250
1:5K
1X HSP60
1:1K
2XR
1:5K.

Figure 2A

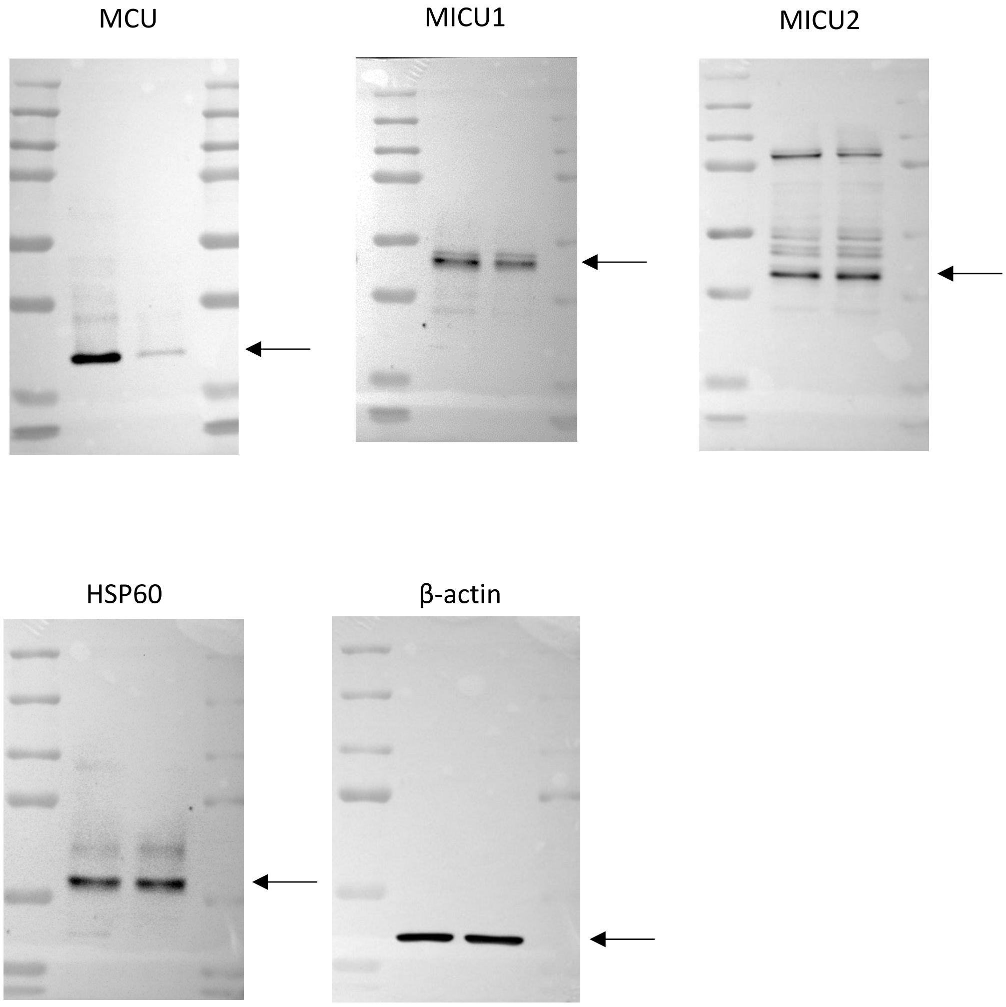

Figure 3B

shScr | shmcu
D0 D2 D5 | D0 D2 D5

MHC

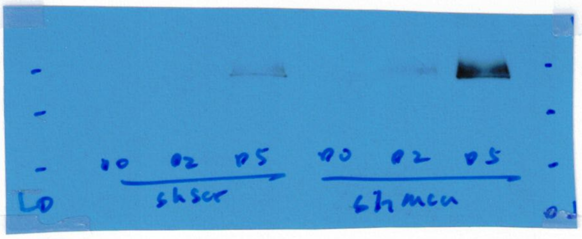

β-actin

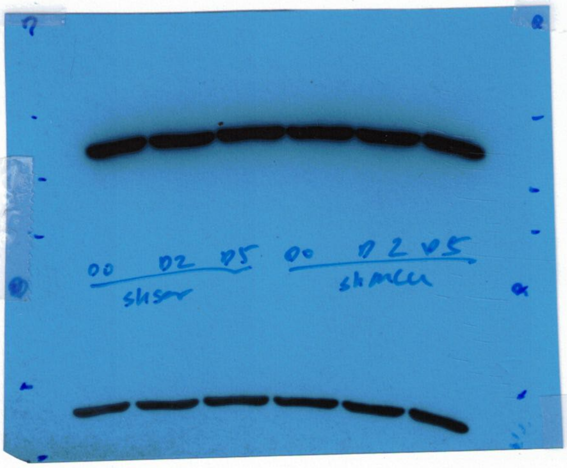

D0 | D2
shScr shmcu | shScr shmcu

MYOG

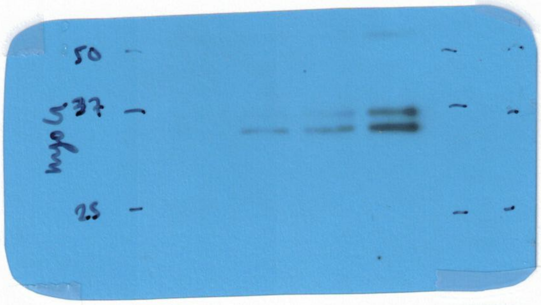

β-actin

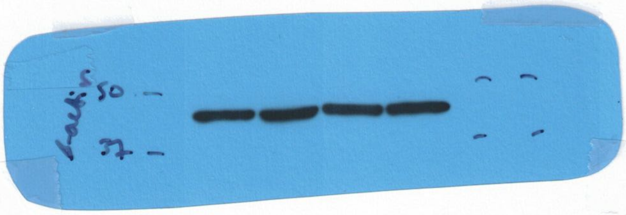

Figure 4F

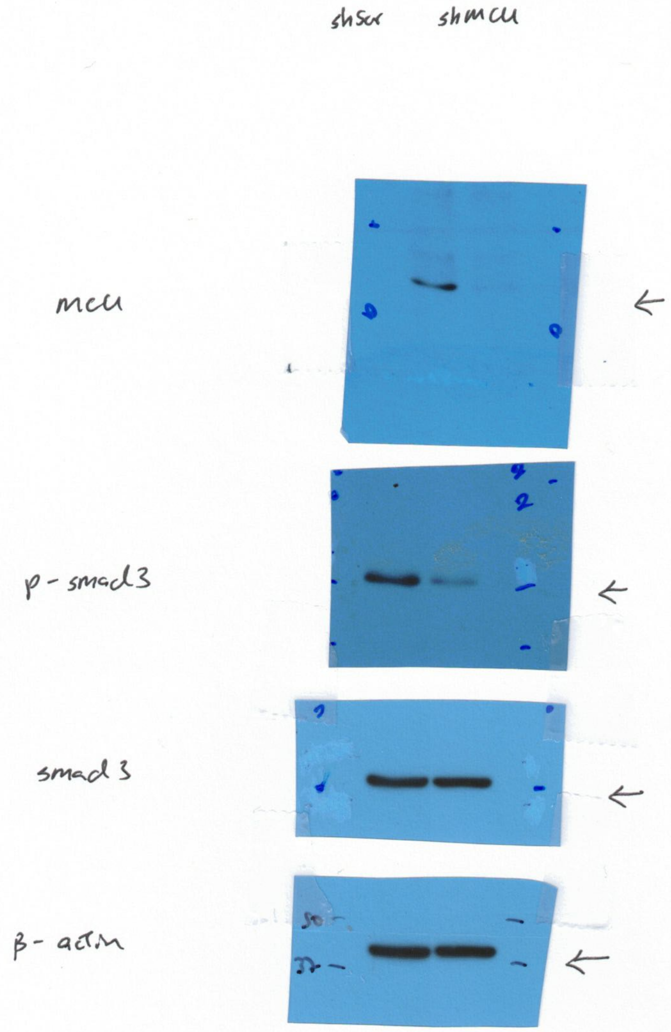

Figure. 5B

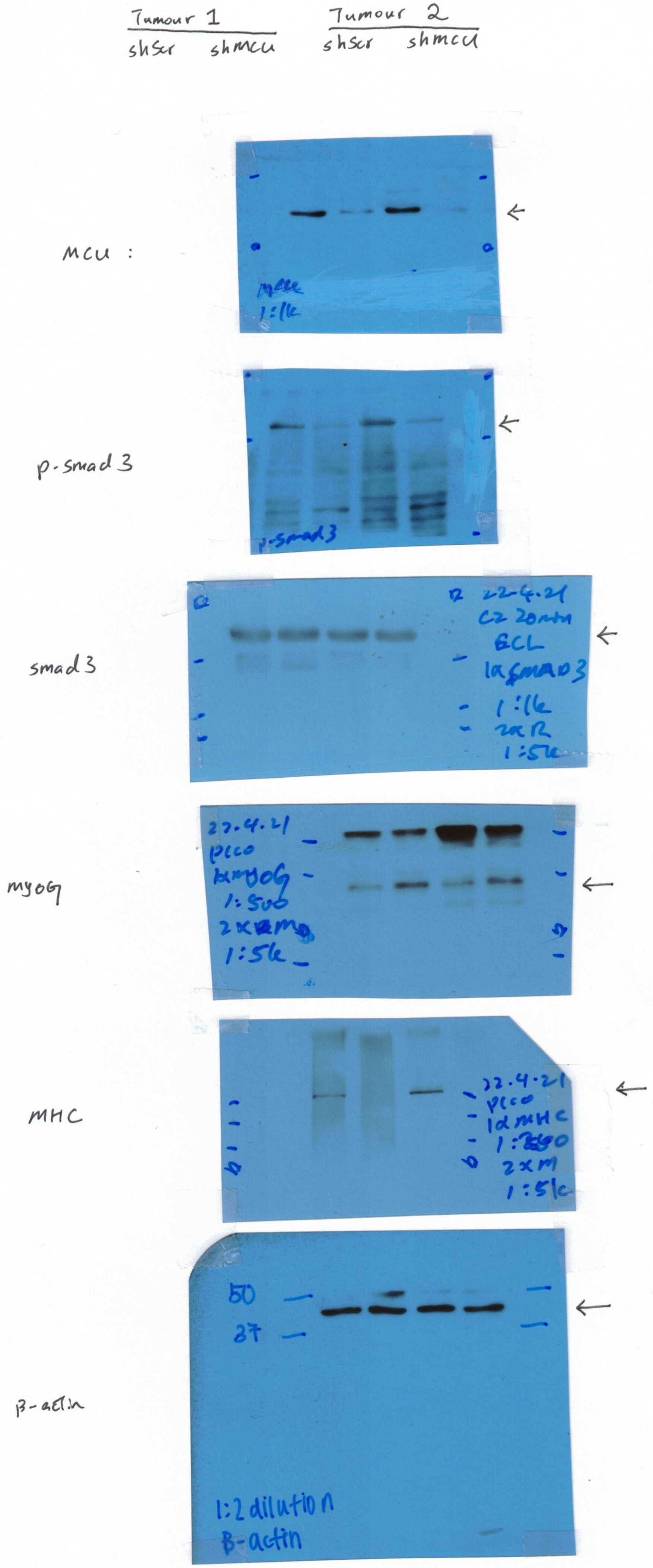
Tumour 1
Tumour 2
shScr
shmcu
shScr
shmcu
MCU :
1:1k
p-smad3
p-smad3
smad3
22-4-21
C2 20min
ECL
1α smad3
1:1k
2αR
1:5k
myoG
22.4.21
pico
1:500
1:5k
MHC
22.4.21
pico
1α MHC
2α M
1:5k
β-actin
50
37
1:2 dilution
β-actin

Figure 6

shScr | shMCU
DMSO mT AA | DMSO mT AA

Figure 6C:

p-smad3

smad3

β-actin

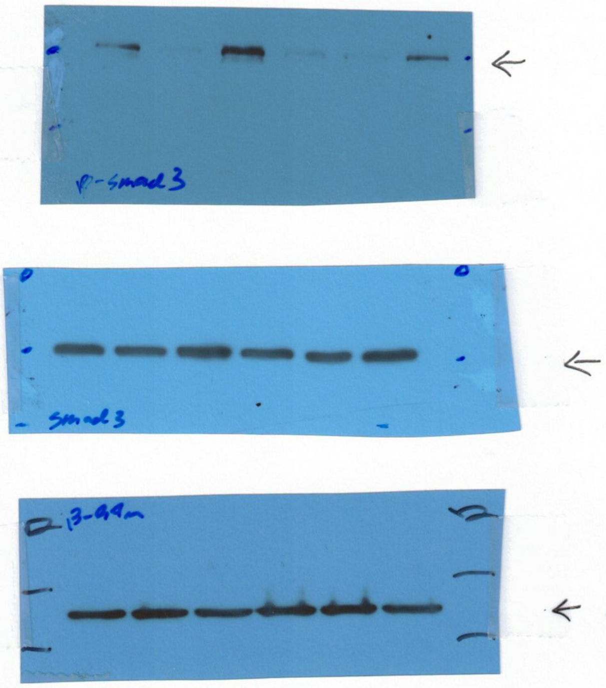

shScr | shMCU
DMSO mT | DMSO AA

Figure. 6E:

myH

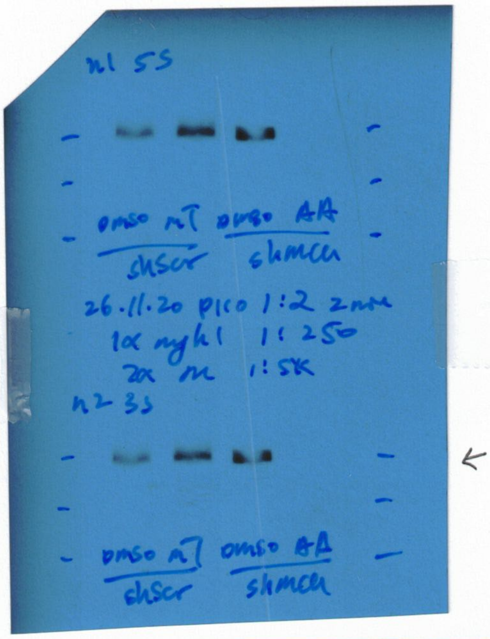

β-actin

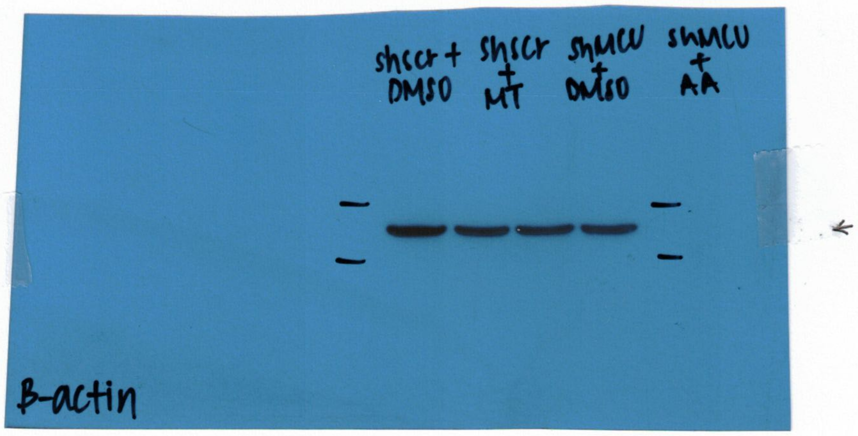

Supplementary Fig. 2A

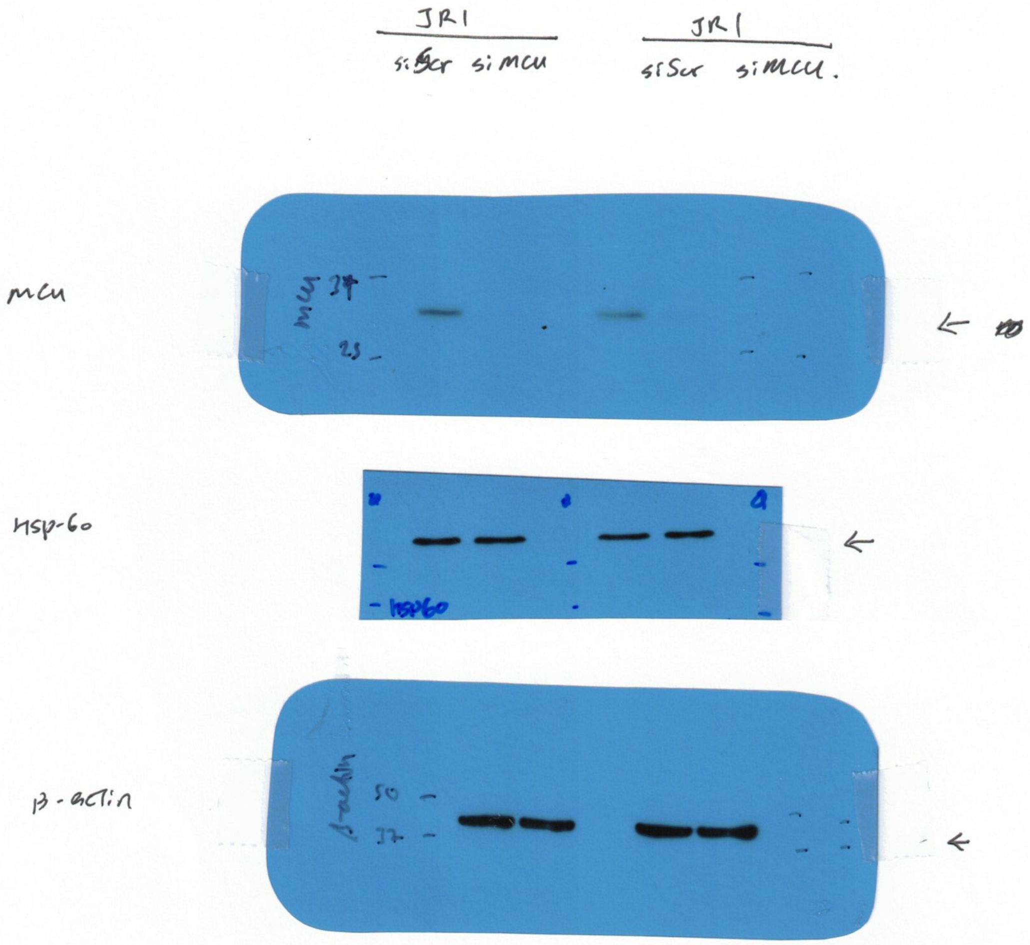

Supplementary Figure 3

Supplementary Figure 3A:

RD

siScr siMCU

MCU

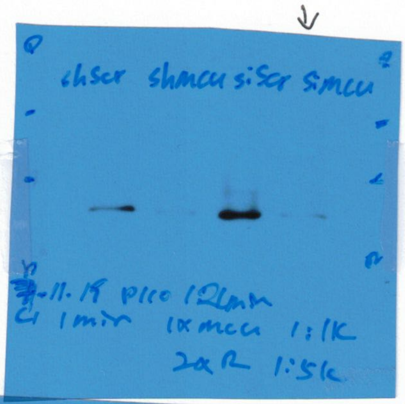

β-actin.

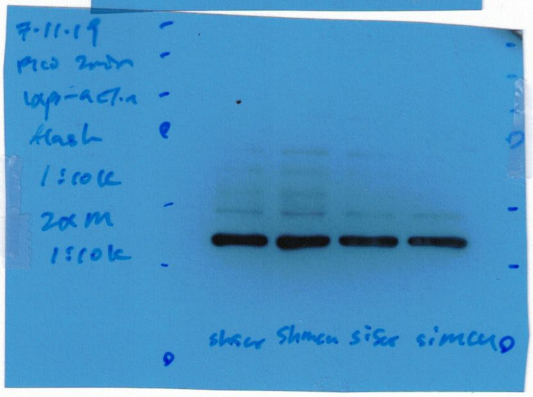

Supplementary Figure 3B&C:

RD18 | JR1
siScr siMCU | siScr siMCU

MCU

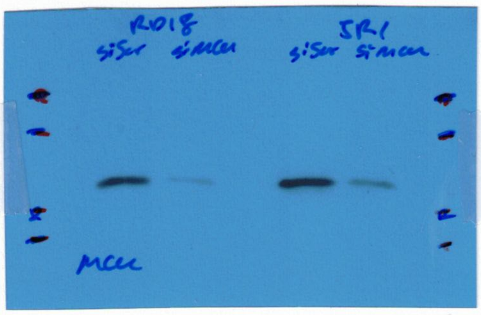

β-actin

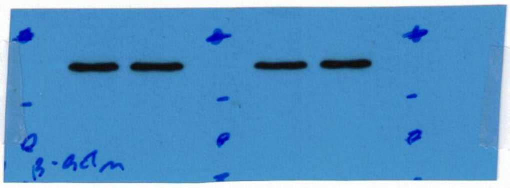

Supplementary Fig. 4

Supplementary Figure 4A:

si Scr | siMCU
D0 D2 D5 | D0 D2 D5

myH

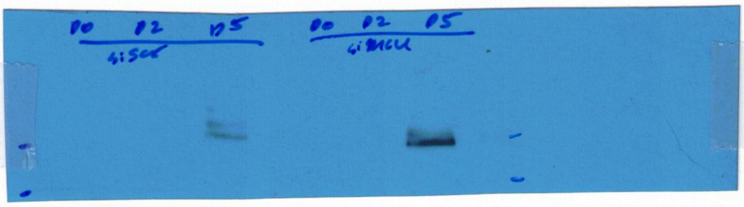

β-actin

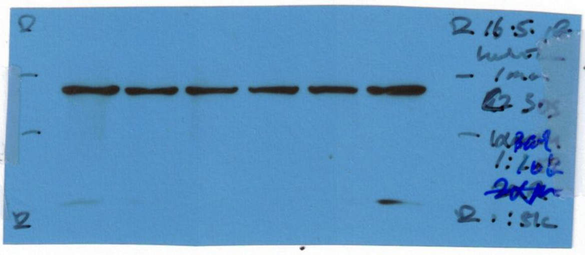

Supplementary Figure 4B:

myH

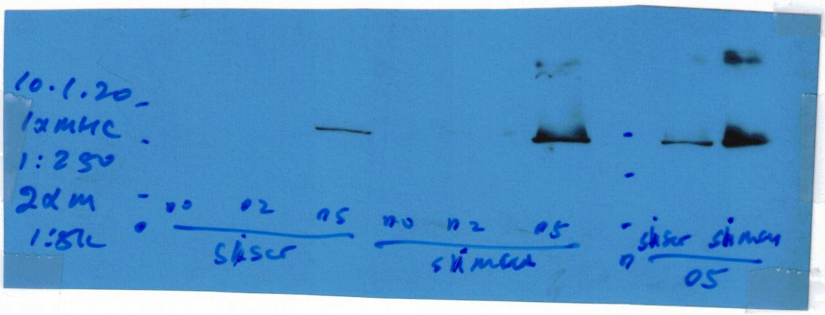

β-actin

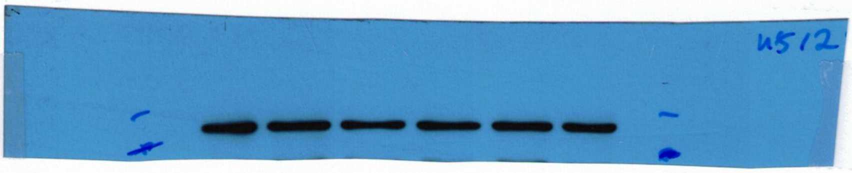

Supplementary Figure 4C:

myH

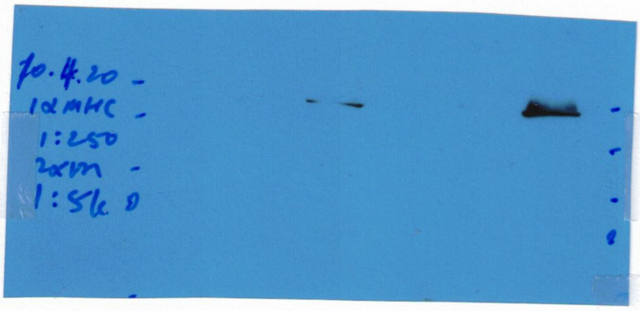

β-Actin

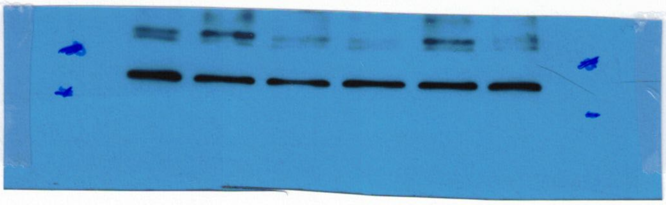

Supplement: Supplementary file 3 — Original Data File [file 41419_2022_4835_MOESM3_ESM.pdf]
