# Supplementary figures and images for: Mitochondrial calcium uptake regulates tumour progression in embryonal rhabdomyosarcoma
Source: Cell Death Dis. 2022 Apr 30;13(4):419. doi: 10.1038/s41419-022-04835-4 (PMC9056521; doi:10.1038/s41419-022-04835-4)

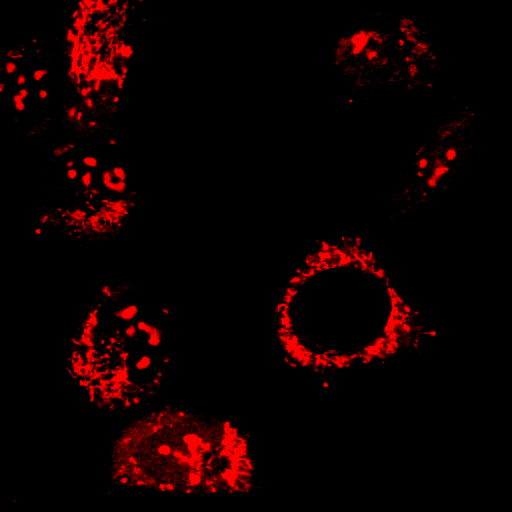

Supplement: Supplementary file 4 — Original Data File [file 41419_2022_4835_MOESM4_ESM.tif]

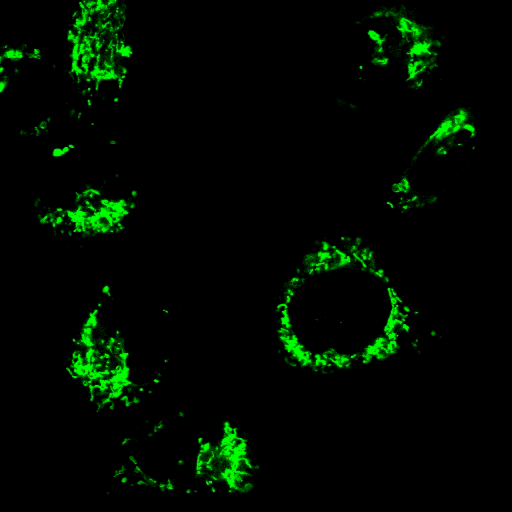

Supplement: Supplementary file 5 — Original Data File [file 41419_2022_4835_MOESM5_ESM.tif]

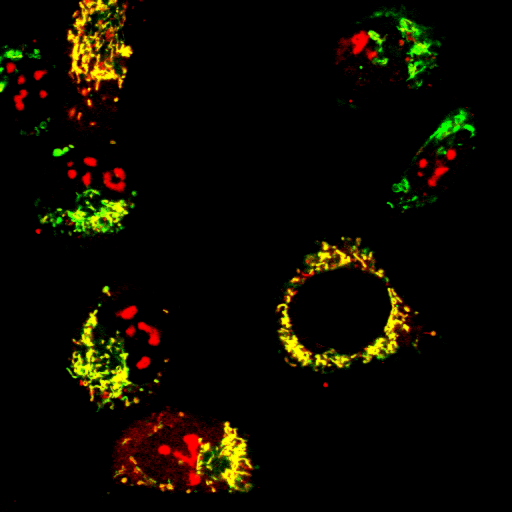

Supplement: Supplementary file 6 — Original Data File [file 41419_2022_4835_MOESM6_ESM.tif]
